# CALDERA: finding all significant de Bruijn subgraphs for bacterial GWAS

**DOI:** 10.1093/bioinformatics/btac238

**Published:** 2022-06-27

**Authors:** Hector Roux de Bézieux, Leandro Lima, Fanny Perraudeau, Arnaud Mary, Sandrine Dudoit, Laurent Jacob

**Affiliations:** Pendulum Therapeutics, Inc., San Francisco, CA 94107, USA; European Bioinformatics Institute, Cambridge CB10 1SD, UK; Pendulum Therapeutics, Inc., San Francisco, CA 94107, USA; Univ. Lyon, Université Lyon 1, CNRS, Laboratoire de Biométrie et Biologie Évolutive UMR 5558, Villeurbanne 69100, France; Division of Biostatistics, Department of Statistics, University of California, Berkeley, CA 94704, USA; Univ. Lyon, Université Lyon 1, CNRS, Laboratoire de Biométrie et Biologie Évolutive UMR 5558, Villeurbanne 69100, France

## Abstract

**Motivation:**

Genome-wide association studies (GWAS), aiming to find genetic variants associated with a trait, have widely been used on bacteria to identify genetic determinants of drug resistance or hypervirulence. Recent bacterial GWAS methods usually rely on *k*-mers, whose presence in a genome can denote variants ranging from single-nucleotide polymorphisms to mobile genetic elements. This approach does not require a reference genome, making it easier to account for accessory genes. However, a same gene can exist in slightly different versions across different strains, leading to diluted effects.

**Results:**

Here, we overcome this issue by testing covariates built from closed connected subgraphs (CCSs) of the de Bruijn graph defined over genomic *k*-mers. These covariates capture polymorphic genes as a single entity, improving *k*-mer-based GWAS both in terms of power and interpretability. However, a method naively testing all possible subgraphs would be powerless due to multiple testing corrections, and the mere exploration of these subgraphs would quickly become computationally intractable. The concept of testable hypothesis has successfully been used to address both problems in similar contexts. We leverage this concept to test all CCSs by proposing a novel enumeration scheme for these objects which fully exploits the pruning opportunity offered by testability, resulting in drastic improvements in computational efficiency. Our method integrates with existing visual tools to facilitate interpretation.

**Availability and implementation:**

We provide an implementation of our method, as well as code to reproduce all results at https://github.com/HectorRDB/Caldera_ISMB.

**Supplementary information:**

Supplementary data are available at *Bioinformatics* online.

## 1 Introduction

Genome-wide association studies (GWAS) look for genetic variants whose presence or absence is associated with a trait of interest, such as the risk for a person to develop a disease, or the yield for a crop. They were originally used on human genomes using single-nucleotide polymorphisms (SNPs) as genetic variants (Visscher *et al.*, 2017). While SNPs do capture most of the genetic variation in genomes that are *similar enough*, they can miss essential variants in other situations. For example, some bacterial species are known to have large accessory genomes, i.e. sets of genes that are not present in every strain in the species. In spite of their name, some of these accessory genes play a central role for some traits of interest, such as antibiotic resistance. In *Pseudomonas aeruginosa*, for instance, accessory genes account for 70% of known genetic determinants of resistance to amikacin (Jaillard *et al.*, 2017). In this context, *k*-mers—defined as all words of length *k* found in the genomes—have emerged as a popular alternative to SNPs to describe genetic diversity (Earle *et al.*, 2016; Sheppard *et al.*, 2013). More specifically, bacterial GWAS often test the association between the trait of interest and the presence/absence of *k*-mers. A broad variety of genetic variants—ranging from SNPs to mobile genetic elements or translocations—cause the mutated strains to contain one or several specific *k*-mers. These GWAS are therefore able to capture any of these variants without requiring their prior identification or even definition. On the other hand, *k*-mer-based GWAS suffer from two important limitations. First, interpreting their result is notoriously tedious: any given *k*-mer can belong to several regions of the same genome, and conversely a gene causing the trait of interest can contain many specific *k*-mers. Second, because a resistance-causing gene often exists in slightly different version, the *k*-mers of each version are only present in a fraction of the resistant strains. As a consequence, these *k*-mers are less strongly associated with resistance than the presence of the polymorphic gene itself.

**Fig. 1.**
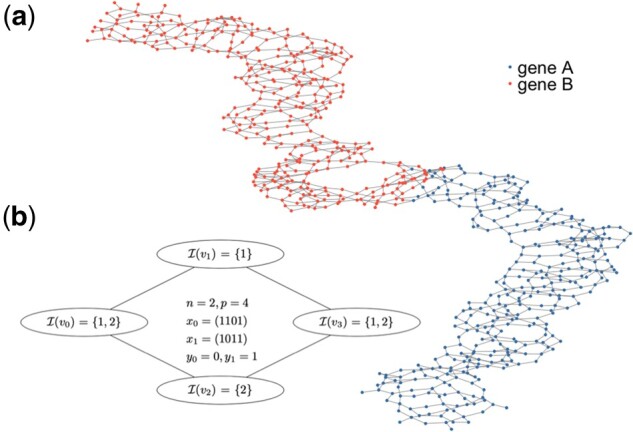
Example of de Bruijn graphs. (**a**) A general example with two genes, each with some variability, resulting in a mostly linear sequence only at the coarse level. More details in Section 4. (**b**) A simpler setting with two samples and four nodes, leading to three CCSs: $\left\{v_{1}\},\;\{v_{2}\right\}$ and $\left\{v_{0},v_{1},v_{2},v_{3}\right\}$

Jaillard *et al.* (2018) proposed DBGWAS to help interpret the result of *k*-mer-based GWAS using the de Bruijn graph (DBG, de Bruijn, 1946; Pevzner *et al.*, 2001), which connects overlapping *k*-mers. Several significant *k*-mers arising from a single polymorphic gene typically aggregate into a somewhat linear subgraph of the DBG (Fig. 1), making their interpretation easier. Similarly, pyseer (Lees *et al.*, 2018), a widely-used bacterial GWAS pipeline, now recommends using unitigs over *k*-mers. However, DBGWAS still tests the individual nodes of this subgraph separately, at the risk of missing causal genes whose presence is too diluted across different versions and therefore different *k*-mers. Kover (Drouin *et al.*, 2016) uses conjunction and disjunction of patterns of presence/absence of *k*-mers to predict the phenotype. However, that approach does not directly allow performing inference and requires specifying the maximum number of allowed combinations.

Here, we propose to test the association between the phenotype and a single covariate capturing the presence of any version of a gene—or any other potential genetic determinant. Concretely, this covariate indicates the presence in each genome of any *k*-mer among those represented in a particular connected subgraph of the DBG. More specifically, we restrict ourselves to closed connected subgraphs (CCSs). A CCS is a connected subgraph such that adding any neighbor does not change the created covariate (i.e. the set of samples containing a *k*-mer that is also in the subgraph). Non-closed subgraphs are represented by the exact same covariate as their closure, and would therefore be redundant.

As any CCS may represent a causal variant that exists in several versions in the dataset, we take an agnostic approach and test the association between the phenotype and one covariate for every CCS in the DBG. In contrast, DBGWAS relies on one covariate for each node of the DBG. This new approach has two potential issues: (i) the number of CCSs grows exponentially with the number of nodes in the DBG, making the task computationally intractable, and (ii) adjusting for multiple testing over this very large number of tests leaves little to no power to detect associations. Our method addresses these two issues by using the concept of testability introduced by Tarone (1990). Tarone’s procedure controls the family-wise error rate (FWER) while disregarding numerous *non-testable* hypotheses in its multiple testing correction. Intuitively, a covariate representing the presence of any *k*-mer among a growing set that corresponds to larger and larger CCS quickly becomes true for all samples. It thus cannot possibly be associated to any phenotype and can therefore be discarded without being tested or counted toward multiple testing correction. Testability provides a well-grounded and quantitative version of this intuition. Furthermore, since adding nodes to a connected subgraph can only increase the number of present *k*-mers in the corresponding covariate, we can develop a method that rapidly prunes non-testable CCSs, thereby solving the computational problem.

Testability has been used in similar situations, but most existing procedures are restricted to complete (Minato *et al.*, 2014; Terada *et al.*, 2013) or linear graphs (Llinares-López *et al.*, 2015, 2017). Sese *et al.* (2014) described an algorithm to test all CCSs by combining the testability-based procedure LAMP of Terada *et al.* (2013) with COIN (Sese *et al.*, 2010), an enumeration method for CCSs. While no experiment was provided in Sese *et al.* (2014), we show that a version of this algorithm using an improved version of LAMP (Llinares-López *et al.*, 2015; Minato *et al.*, 2014) could find all significant CCSs in graphs with up to 20 000 nodes in less than a day in only the most favorable settings. However, the DBG built for typical bacterial GWAS involve millions of nodes, so a more scalable method is necessary to make CCSs testing amenable.

*Our contributions are the following*: We introduce a novel, provably complete and non-redundant enumeration scheme for CCSs called CALDERA. We also improve an existing pruning criterion for the Cochran–Mantel–Haenszel (CMH) test. We show that combining these contributions with Tarone’s testability-based procedure makes it possible to find all significant CCSs in a large graph, making it suited to bacterial GWAS. We provide the first implementation of a procedure finding all significant CCSs, along with a user-friendly visualization tool derived from DBGWAS. Finally, we demonstrate the advantages of CALDERA over competing methods on both simulated and real examples in terms of computational speed, statistical power and biological interpretation.

*Notation and goal for CALDERA*: We consider a set of *n* samples, ${\left(x_{i},y_{i},c_{i}\right)}_{i=1}^{n}$, where $x_{i}\in {\left\{0,1\right\}}^{p}$ are *p* binary covariates describing sample *i*, $y_{i}\in \{0,1\}$ denotes a binary phenotype, and $c_{i}\in \{1,\ldots ,J\}$ assigns sample *i* to one population among *J*. We denote *n*_1_ and *n*_2_ the number of samples such that *y_i_* = 0 and 1, respectively. Furthermore, we consider an undirected unweighted connected graph G=(V,E), where $V=\{v_{1},\ldots ,v_{p}\}$ and each vertex $v_{j}\in V$ is associated with one of the *p* binary covariates represented in *x*. We denote by $I(v_{j})=\{i:x_{i}^{j}=1\}$ the indices of samples containing *v_j_*. Conversely, for i∈[1:n], we note $V_{i}=\{v\in V:i\in I(v)\}$ the set of covariates contained by the *i*-th sample. For any connected subgraph S=(V′,E′), such that V′⊆V and E′⊆E, we let $I(S)=\underset{v\in V\prime }{\cup }I(v)$ be the indices of samples containing at least one of the covariate represented by the vertices of S. Of note, this framework addresses both disjunctions and conjunctions, as the latter can simply be obtained by replacing each *x_i_* by its complement. We now properly define the notion of CCS and the closure operation (proof in Supplementary Section S-1.1).

Definition 1. *A connected subgraph* S  *is closed if and only if there exists no edge* $(v_{1},v_{2})\in E$  *such that* $v_{1}\in S$, $v_{2}\notin S$*, and* $I(S\cup \left\{v_{2}\right\})=I(S)$*. We denote by* C  *the set of all closed connected subgraphs of* G.

Lemma 1. *For any connected subgraph* S  *of* G*, there exists a unique subgraph* S′∈C  *such that* I(S)=I(S′)  *and* S⊆S′*, which we note* cl(S).

Assuming that ${\left(x_{i},y_{i},c_{i}\right)}_{i=1}^{n}$ are *n* i.i.d. realizations of random variables X,Y, and **C**, our objective is to test null hypotheses of the form $H_{0}^{S}(X,Y,C):(I(S)⊥Y)|C$ for all S∈C, while controlling the FWER (i.e. the chance of at least one Type I error or false positive) at level *α*. Translated in the context of GWAS, we want to test the association between the phenotype **Y** and the presence pattern I(S) of the covariate represented by each CCS S, while controlling for the population structure **C**. We denote $H_{0}(S)=H_{0}^{S}(X,Y,C)$ in the remainder of this manuscript, as **X**, **Y** and **C** are common for all CCS in C.

## 2 Background on significant subgraph detection using testability

We now describe the important concept of minimal attainable *p*-value proposed by Tarone (1990), and how it can be used to (i) retain more power than the Bonferroni procedure while controlling the FWER and (ii) test more rapidly a large set of hypotheses. Both improvements come from the possibility to discard a large proportion of hypotheses without explicitly testing them, and will be exploited in Section 3 to propose our procedure testing all CCSs in C.

Minimal *p*-values are a property of discrete tests. For example, Fisher’s exact test (Fisher, 1922) relies on a 2 × 2 contingency table, whose margins would describe in our case the number of sensitive and resistant bacteria and the number of bacteria whose genome contains or not a genetic variant. Given the margins of this table, only a finite number of cell count assignment are possible and Fisher’s test can only lead to a finite number of values, the smallest of which is strictly positive (see Supplementary Fig. S1 for an example). Importantly, this minimal attainable *p*-value $p^{⋆}$ is entirely determined by the margins of the contingency table: given these margins, $p^{⋆}$ is the minimum over a finite number of possible partitions and is independent of the actual observed cell counts. Intuitively, strongly imbalanced margins (e.g. variants that are present in a very large proportion of samples) cannot possibly lead to small *p*-values, no matter how the table is filled (i.e. how the few samples that do not have the variant are distributed among resistant and sensitive phenotypes).

### 2.1 Using minimal attainable *p*-values for a tighter FWER control

The FWER is the probability to incorrectly reject at least one null hypothesis. When testing *N* of them and rejecting those whose *p*-value *p_i_* is smaller than a threshold *δ*, $\text{FWER}(\delta )=P\left({⋁}_{i=1}^{N}\left(p_{i}\leq \delta \right)\right)$, where P is taken over the *N* null distributions ${\left(H_{0}^{i}\right)}_{i=1}^{N}$. The Bonferroni correction (Bonferroni, 1936) is a common procedure to control the FWER at a level *α*. It is motivated by a simple union bound: as FWER(δ) is upper-bounded by $\sum_{i=1}^{N}P_{H_{0}^{i}}\left(p_{i}\leq \delta \right)$ and since by definition $P_{H_{0}^{i}}\left(p_{i}\leq \delta \right)\leq \delta$, controlling each individual tests at level $\delta =\frac{\alpha }{N}$ makes the FWER upper-bounded by *α*. Tarone (1990) sharpens this bound, by using the fact that $p_{i}^{⋆}>\frac{\alpha }{N}$ for some hypotheses. Since by definition $p_{i}\geq p_{i}^{*}$, the corresponding $P\left(p_{i}\leq \frac{\alpha }{N}\right)$ term is exactly 0. Therefore, the FWER is actually controlled at level $\frac{m\alpha }{N}\leq \alpha$ where *m* is the number of testable hypotheses, for which $p_{i}^{⋆}\leq \frac{\alpha }{N}$. This suggests that using a larger threshold *δ* than the Bonferroni $\frac{\alpha }{N}$ could still control the FWER at level *α*—while rejecting more hypotheses and therefore increasing power. Choosing the largest such *δ* is not a trivial task, as increasing *δ* also increases the number *m* of non-testable hypotheses. Let *m*(*k*) be the number of testable hypotheses at level $\frac{\alpha }{k}$, i.e. such that $p^{⋆}<\frac{\alpha }{k}$. In the worst case, *m*(*k*) = *N* and we recover the Bonferroni procedure. More generally, Tarone’s analysis guarantees that FWER(δ)≤α for $\delta =\frac{\alpha }{k}$ as long as m(k)≤k. Letting *k*_0_ be the smallest *k* verifying this property, $\delta =\frac{\alpha }{k_{0}}$ maximizes the number of rejections while controlling the FWER at level *α*.

### 2.2 Using minimal *p*-values to efficiently explore C

Provided that enough CCSs have sufficiently large $p^{⋆}$, Tarone’s procedure could therefore address the loss of power incurred when exploring C. However, naively finding *k*_0_ requires computing the minimal *p*-values for all |C| CCSs and iterate through these minimal *p*-values to adjust the threshold, leaving the computational problem unsolved. A more efficient strategy has been introduced to compute *k*_0_ (Llinares-López *et al.*, 2015; Minato *et al.*, 2014): starting from *k *=* *1 a set R of *testable* hypotheses, i.e. of elements with $p^{⋆}<\frac{\alpha }{k}$ is grown. When |R| becomes larger than *k*, *k* is incremented to |R|. All hypotheses that are not testable anymore under the new threshold—i.e. such that $\frac{\alpha }{\left|R\right|}\leq p^{⋆}<\frac{\alpha }{k}$ —are removed from |R|, and the exploration continues until the point where all testable hypotheses are in R and *k* = *k*_0_. This strategy finds *k*_0_ in a single enumeration of all tests, but still requires computing all minimal *p*-values, which would not be feasible in our case. However, this search algorithm is also well suited to pruning strategies—a fact already used in Llinares-López *et al.* (2015) and Minato *et al.* (2014). Let $p^{⋆}(S)$ be the minimal *p*-value associated with $H_{0}(S)$ for a CCS S. Assuming that for some pairs of subgraphs $S_{1},S_{2},\;S_{1}\subseteq S_{2}\Rightarrow p^{⋆}(S_{1})\leq p^{⋆}(S_{2})$, we can stop exploring all subgraphs including $S_{1}$ as soon as $S_{1}$ itself is found non-testable. This monotonic property is verified when using Fisher’s exact test to test $H_{0}(S)$: provided that $|I(S)|\geq \max(n_{1},n_{2}),\;p^{⋆}$ is strictly increasing in |I|, and adding nodes to S can only increase |I| (see Supplementary Fig. S2 for an example). Our main contribution, presented in Section 3 will be an efficient exploration algorithm for S, which is well suited to pruning.

### 2.3 Controlling for a categorical covariate: the CMH test

When testing for associations, controlling for confounders is essential to avoid spurious discoveries. This is particularly important in bacterial GWAS, where strong population structures can lead to large sets of clade-specific variants to be found associated with a phenotype. The CMH test can be used to test associations of two binary variables while controlling for a third categorical variable. It relies on *J* two-by-two association tables such as the one in Table 1, with $j\in \{1,\ldots ,J\},\;a_{S,j}=|\{i:y_{i}=1,i\in I(S),c_{i}=j\}|,\;x_{S,j}=|\{i:i\in I(S),c_{i}=j\}|$ and $n_{1,j}=|\{i:y_{i}=1,c_{i}=j\}|$.

**Table 1.** Association table in community *j* for subgraph S, used for the CMH test

Variable	i∈I(S)	i∉I(S)	Rows totals
$y_{i}=1$	$a_{S,j}$	$n_{1,j}-a_{S,j}$	$n_{1,j}$
$y_{i}=0$	$x_{S,j}-a_{S,j}$	$n_{2,j}-x_{S,j}+a_{S,j}$	$n_{2,j}$
**Cols totals**	$x_{S,j}$	$n_{j}-x_{S,j}$	*n_j_*

Like Fisher’s exact test, the CMH test is done conditional on all margins ${\left(x_{S,j},n_{1,j},n_{2,j}\right)}_{j=1}^{J}$. Papaxanthos *et al.* (2016) furthermore, demonstrated that its minimal *p*-value could be computed in *O*(*J*) (proof in Supplementary Section S-1.5) using the margins. However, the minimal *p*-value of the CMH test does not verify the monotonicity property $S_{1}\subseteq S_{2}\Rightarrow p^{⋆}(S_{1})\leq p^{⋆}(S_{2})$ which is required to prune while exploring C. Papaxanthos *et al.* (2016) introduced the envelope, a lower bound on $p^{⋆}(S)$, which verifies the monotonicity property. It can also be computed in O(J log(J)) for all S such that, for all categories *j*, $x_{S,j}\geq \max(n_{1,j},n_{2,j})$. This allows for a valid pruning strategy. The condition on $x_{S,j}$ is the CMH analogous of the $\left|I(S)|\geq \max(n_{1},n_{2}\right)$ condition of Fisher’s test, and can decrease the number of prunable subgraphs as it must be verified for all *J* groups.

## 3 Speeding up the detection of all significant CCSs with CALDERA

We are now ready to present our contributions for scalable detection of significant elements in C: an efficient exploration algorithm and an improved envelope for the CMH test, allowing for more pruning in the presence of imbalanced populations.

### 3.1 Critical properties for a fast, Tarone-aware enumeration of C

We exploit several factors to provide a fast exploration of C. First, we ensure that it is non-redundant, i.e. that each element of C is enumerated exactly once, by defining a tree whose nodes are the elements of C and propose an algorithm to traverse this tree. Second, the tree is directly built over C, as opposed to the set of connected subgraphs. The latter option, as proposed in Sese *et al.* (2010) is more straightforward to define and to explore and still induces a tree over C, but yields a much larger object and results in a more expensive traversal. Third, we avoid maintaining subgraph connectivity, such as a block-cut tree (Westbrook and Tarjan, 1992). Such a mechanism is efficient to build a tree over connected subgraphs, but is costly to compute. Finally, in order to exploit the pruning opportunity offered by the testing procedure, the exploration should be such that all S′ explored from a given S verify S′⊃S.

Haraguchi *et al.* (2019) and Okuno *et al.* (2017) define a tree on C, but the root of the tree corresponds to the entire graph G: the inclusion relationship along edges of the tree is the opposite to the one we need, making their exploration unsuited to our problem. The COIN/COPINE algorithm described in Seki and Sese (2008); Sese *et al.* (2010) builds a tree over the set of connected subgraphs, which induces a tree over C but has two drawbacks. First, it maintains an itemtable to avoid enumerating the same element twice. This itemtable has an important memory footprint, and only guarantees a tree structure when exploring in depth first. Secondly, the enumeration of connected subgraphs requires maintaining a list of articulation points along each explored branch, a costly operation.

**
Algorithm 1 Children of $S_{p}$**

**Input** parent CCS $S_{p}$, current CCS S, largest index *i*, itemtable T

1: **procedure** Children($S_{p},\;S$, *i*, T)

2:   children←∅

3:   **for** *k*, *G* in enumerate(EqGroups(S)) **do**

4:     S′←cl(S∪{G[0]})  S′ is a candidate child

5:   **if**  $\left(S_{p},S\prime \right)$ verify (C1−3) **then**  S′ is a child

6:     **if** *i* is NULL **then** Exploring from the direct neighbors of $S_{p}$

7:        Add S′ to children

8:      Add Children$\left(S_{p},S\prime ,i_{S\prime },T=\emptyset \right)$ to children

9: **else**   Exploring from the neighbors of another child

10:     **if**  $i_{S\prime }=i$ and {I∈T:I⊂I(S′)}=∅  **then**     Check that S′ was not enumerated earlier

11:        $T\prime =T\cup \{I_{1}(S),\ldots ,I_{k-1}(S)\}$

12:        Add S′ to children

13:        Add Children$\left(S_{p},S\prime ,i_{S\prime },T\prime \right)$ to children

14:       **end if**

15:     **end if**

16:   **end if**

17:   **end for**

18:   **return** children

19: **end procedure**

### 3.2 Defining and exploring the tree over C

In order to build a tree over C rooted on the empty CCS, we use a reverse search, a strategy introduced in Avis and Fukuda (1996). Reverse search relies on a reduction operation, which takes one element of the set to be enumerated, and returns a unique, strictly smaller element of the same set. This operation necessarily defines a tree over the elements of the set, by ensuring a unique path between any element and the empty one—the root of the tree. This reduction operation defines the unique parent of every element in the tree. In order to traverse the tree from the root, one needs to inverse the reduction operation, i.e. in our setting, given a CCS S to recover all CCSs that lead to S by reduction. Here, we introduce a reduction operation over C, as well as its inversion. We consider the parent operation P given by Definition 2 for any element of C, and show that it defines a valid reduction as introduced above. We then show its inversion in Algorithm 1. All proofs are presented in the Supplementary Appendix.

Definition 2. *For a subgraph* S∈C*, we denote* $J(S)=\underset{v\in S}{\cap }I(v)$  *the indices of samples containing all covariates represented by nodes in* S.

- *If* I(S)=J(S)*, then the parent of* S  *is* ∅*, i.e.* P(S)=∅.
- *Else we note* $i_{S}=\max_{i}\{i:i\in I(S)\setminus J(S)\}$*. The parent* P(S)  *of* S  *is the connected subgraph of* $S\setminus V_{i_{S}}$  *that contains* $\max_{v}\{v:v\in S\setminus V_{i_{S}}\}$.

Here, the $\max_{v}$ over a set of nodes is defined through some arbitrary numbering of the elements of V. In layman’s terms, our reduction first finds the largest index $i_{S}$ among samples that contain at least one but not all covariates represented by nodes in S. It then removes all nodes representing a covariate contained by this sample, and retains one of the connected components as P(S). Our reduction therefore relies on a numbering of both the samples—to decide which nodes are removed—and the nodes—to define which connected component resulting from the removal is retained as the unique parent.

Lemma 2. *The function* P  *defines a valid reduction over* C.

Note that we have S⊃P(S) for all S so this structure allows pruning. Lemma 3 then provides necessary and sufficient conditions for S′∈C to be a child of S∈C. The third condition involves the set of neighboring nodes of S, defined as $Ne(S)=\{v\in G\setminus S:\exists v_{1}\in S,(v,v_{1})\in E\}$.

Lemma 3. *For* S,S′∈C  *such that* S⊂S′≠∅*, we have:* S=P(S′)  *if and only if the three following conditions are verified:*

*(C1)* $i_{S\prime }\notin I(S)$

*(C2)* $\max_{v\prime }\{v\prime \in S\prime \setminus V_{i_{S\prime }}\}=\max_{v}\{v:v\in S\}$

*(C3)* $\{v\prime \in S\prime \setminus V_{i_{S\prime }}:v\prime \in Ne(S)\}=\emptyset$*, or written differently*, $(S\prime \setminus V_{i_{S\prime }})\cap Ne(S)=\emptyset$.

Using (C1–3) in Lemma 3 to check whether S=P(S′) for any S′ does not require identifying the connected components of $S\prime \setminus V_{i_{S\prime }}$, even though the reduction P itself does rely on these connected components. This property of the inverse reduction is critical for the scalability of CALDERA: repeatedly identifying or maintaining these components would be very costly. It results from the fact that the reduction operation P does not maintain full connectivity, but only retains one of the connected components obtained by removing a subset of its nodes. Doing so comes at a price: finding all children of S is not straightforward—Lemma 3 only provides a way to check if a candidate S′ is a child of S. We must therefore provide a non-redundant way to explore all potential children, after which Lemma 3 will guarantee a non-redundant exploration of C.

More precisely, reducing any CCS S′ to its parent S involves the removal of a subset $V_{i_{S\prime }}$ of its nodes, breaking S′ into several connected components—the one containing the largest vertex being retained as the unique parent. For this reason, the reverse search formalized in Algorithm 1 cannot just search for children of S among all closures obtained after adding one of its neighbors Ne(S) (Lines 6–7): larger CCSs may also lead to S by reduction if they involve other nodes that are not in its direct neighborhood. For example, the graph shown in Figure 1 and discussed in Section 3.3 contains two CCS ($\left\{v_{1},v_{3},v_{4}\right\}$ and $\left\{v_{1},v_{2},v_{3},v_{4}\right\}$) which both lead to $\left\{v_{3}\right\}$ by reduction but cannot be obtained from $\left\{v_{3}\right\}$ by just adding its (single) neighbor and taking the closure. For every identified child S′—e.g. $\left\{v_{3},v_{4}\right\}$ in the example—we must therefore recursively search for other candidates among the closures obtained after adding one of its own neighbors Ne(S′)—nodes *v*_1_ or *v*_2_ in the example. This procedure is necessary to reconnect all children that include S′ but would leave it as a separated connected component after removing nodes $V_{i_{S\prime }}$—node *v*_4_ in the example. This recursive exploration is guaranteed to visit each candidate child, but does not ensure that each child is visited only once. A redundant exploration would lose the benefit of building a tree to explore C efficiently. We therefore need an itemtable T that keeps track of visited patterns I: if a candidate child S″ has a pattern I(S″) that includes the pattern of an already enumerated child from the neighborhood of the same S′, we know that S″—and any child that could be obtained from it—has already been visited and the algorithm stops exploring from S″. In practice, we do not need to store the full table T, and rely on a concept from Uno *et al.* (2004) and further described in Supplementary Section S-2 to reduce memory footprint.

Theorem 1. *For any* S∈C,  *Algorithm 1 applied on* (S,S,*NULL*,∅)  *returns the set* {S′∈C:S=P(S′)}.

Theorem 1 says that Algorithm 1 solves the problem of inverting the reduction, and therefore of building a tree structure on C. Of note, Algorithm 1 effectively explores equivalence groups of neighbors, yielding the same pattern. Formally, an equivalence group $G_{k}(S)\subset Ne(S)$ verifies: $v_{1},v_{2}\in G_{k}(S)\Rightarrow I(S\cup \left\{v_{1}\right\})=I(S\cup \left\{v_{2}\right\})$. We name $I_{k}(S)$ the pattern of the *k*-th equivalence group $G_{k}(S)$.

### 3.3 Example of exploration

**Fig. 2.**
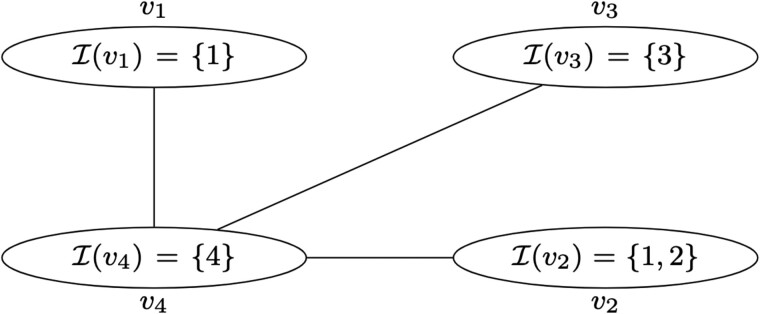
A short illustration of CALDERA’s exploration. A simple graph with four nodes and four samples. Nodes *v*_1_, *v*_2_ and *v*_3_ are linked to node *v*_4_. To construct the CCS $\left\{v_{1},v_{2},v_{3},v_{4}\right\}$ from $\left\{v_{3},v_{4}\right\}$, we can either first add *v*_1_—and thus construct $\left\{v_{1},v_{3},v_{4}\right\}$—and then add *v*_2_, or directly add *v*_2_ and then we get $\left\{v_{1},v_{2},v_{3},v_{4}\right\}$ by closure. To avoid enumerating $\left\{v_{1},v_{2},v_{3},v_{4}\right\}$ twice, we therefore need local itemtables

To help provide a better intuition of Algorithm 1, we use a simple graph with four nodes and four samples in Figure 2 and will unfold how various CCS are explored. The algorithm technically starts from the empty CCS, whose children are in this case $\left\{v_{1}\},\{v_{2}\},\{v_{3}\},\{v_{4}\right\}$, each of them (i) being closed and (ii) leading to the empty set by reduction.

Starting from *v*_4_, we can construct the connected subgraph $\left\{v_{4}\right\}$ whose pattern $I(\{v_{4}\})=\{4\}$. Adding any of its neighbors will change that pattern, so $\left\{v_{4}\right\}$ is a CCS. Can we explore from $\left\{v_{4}\right\}$? We use Algorithm 1 with $S_{p}=S=\{v_{4}\}$, *i* = *NULL* and T=∅. We enumerate over the neighbors of S (Line 3), which are *v*_1_, *v*_2_ and *v*_3_. For example, with *v*_3_, $S\prime =cl(S\cup \left\{v_{3}\right\})=\{v_{3},v_{4}\}$ (Line 4). $i_{S\prime }=\text{max}\{3,4\}=4$ is equal to $i_{S}$ so we do not verify (C1) (Line 5) and we stop there. The same happens with *v*_1_ and *v*_2_. This can be expected. When applying the reduction to any CCS that contain *v*_4_, we will remove all the nodes containing the biggest sample, that is 4 so we will always remove *v*_4_. So no CCS gives $\left\{v_{4}\right\}$ by reduction. Therefore, when inverting the reduction with Algorithm 1, we should find no children of $\left\{v_{4}\right\}$.

If we start from *v*_3_, we begin similarly. $S=\{v_{3}\}$, whose pattern $I(\{v_{3}\})=\{3\}$, is a CCS. If we add its (only) neighbor *v*_4_ (Line 4), we construct again $S\prime =\{v_{3},v_{4}\}$. But this time we have (C1) $i_{S\prime }=4\neq 3=i_{S}$; (C2) $\max\{v\prime :S\prime \setminus V_{4}\}=\max\{v_{3}\}=\{v_{3}\}=\max\{v\in S\}$; (C3) $S\prime \setminus V_{4}=\{v_{3}\}$ while $Ne(S)=\{v_{4}\}$ so $(S\prime \setminus V_{4})\cap Ne(S)=\emptyset$. So we verify (C1–3), which ensures that $\left\{v_{3},v_{4}\right\}$ is indeed a child of {3} (Line 5).

Since *i* is NULL (Line 6), we add S′ to the list of the children of $S_{p}=\{v_{3}\}$ (Line 7). We then call Algorithm 1 again (Line 8), with $S_{p}=S,\;S\prime =\{v_{3},v_{4}\},\;i=i_{S\prime }=4$ and T=∅. We have two possible neighbors (and corresponding equivalence groups), *v*_1_ and *v*_2_ (Line 3). We first add *v*_2_. However, $\left\{v_{2},v_{3},v_{4}\right\}$ is not closed. By the closure operation, we add *v*_1_ as well, so we have a new $S\prime =\{v_{1},v_{2},v_{3},v_{4}\}$ (Line 4), and $\left(S_{p},S\prime \right)$ verify (C1–3) (Line 5). *i *=* *4 is not NULL anymore, so we move to Line 10. $i_{S}^{\prime }=4=i$ and T=∅. Since *k *=* *1 (we are exploring the first equivalence group from $\left\{v_{3},v_{4}\right\}$), we do not update T′ (Line 11) but add S′ to the children of $S_{p}=\{v_{3}\}$ (Line 12). $S\prime =\{v_{1},v_{2},v_{3},v_{4}\}$ has no neighbors since it is the full graph, so Line 13 returns no more values.

We return to Line 3 to explore the second equivalence group from $\left\{v_{3},v_{4}\right\}$, with *k *=* *2: we add *v*_1_. The new $S\prime =\{v_{1},v_{3},v_{4}\}$ is a CSS (Line 4) and $\left(S_{p},S\prime \right)$ verify (C1–3) (Line 5). *i *=* *4 is not NULL and T=∅ (Line 10). We now update it to $T\prime =T\cup I_{1}(S)=\{I(v_{2})\}=\{\{1,2\}\}$. We add $\left\{v_{1},v_{3},v_{4}\right\}$ to the list of children and call Algorithm 1 again (Line 13), with $S_{p}=\{v_{3}\},\;S\prime =\{v_{1},v_{3},v_{4}\}$, *i *=* *4 and T={{1,2}}.

$\left\{v_{1},v_{3},v_{4}\right\}$ has a single neighbor (Line 3), *v*_2_. $\left\{v_{1},v_{2},v_{3},v_{4}\right\}$ is a CCS (Line 4), $\left(S_{p},\{v_{1},v_{2},v_{3},v_{4}\}\right)$ verify (C1–3) (Line 5). *i* is not NULL. However, I(S′)={1,2,3,4} so {I∈T:I∈I(S′)}≠∅. We stop the exploration here. This illustrates the importance of the local itemtables since without them, we would have enumerated $\left\{v_{1},v_{2},v_{3},v_{4}\right\}$ twice.

Algorithm 1 applied to $\left(\{v_{3}\},\{v_{3}\},\mathrm{NULL},\mathrm{NULL}\right)$ returned $\left\{v_{3},v_{4}\},\{v_{1},v_{2},v_{3},v_{4}\},\{v_{1},v_{3},v_{4}\right\}$. When doing the reduction of any of those CCS, we remove *v*_4_ which breaks the CCS into several components. Since *v*_3_ will always be the biggest remaining node, it will always be picked as the parent. So, when inverting the reduction, we find all subgraphs containing *v*_3_ and *v*_4_ as children.

The remaining CCS will be found similarly by starting from *v*_1_ (for $\left\{v_{1},v_{4}\right\}$) and *v*_2_ (for $\left\{v_{2},v_{4}\right\}$ and $\left\{v_{1},v_{2},v_{4}\right\}$).

### 3.4 A Breadth-first-search (BFS) enumeration

We argue that exploring any tree structure on C in breadth first will often allow for more pruning than in depth first. Pruning occurs among children, not siblings. At any level, even if the CCSs visited along a branch do increase *k* and therefore lower the testability threshold, all the other CCSs of the level will need to be visited regardless of their testability. In contrast, the increase of *k* gained by visiting all CCSs of the same level in the tree will lower the threshold α/k for all CCSs at the next level, making more branches prunable. We demonstrate this in Section 4 and provide more intuitive examples in the Supplementary Appendix (Supplementary Sections S-5.1 and S-5.2). A search in breadth is also easily parallelized since the computation of the minimal *p*-value, the envelope and the children of every CCS of a given level can be done in parallel, before increasing *k* and updating R. In contrast, a parallelized search in depth-first must share and regularly update *k* and R, which negates the advantages of parallelization.

**Algorithm 2 List significant closed connected subgraphs**

1: **procedure** List_sig_closed_subgraphs(G,α)

2:   Q←Children(∅,∅,NULL,∅)

3:   R←∅

4:   k←1

5:   **while**  Q≠∅  **do**

6:     S←Dequeue(Q)

7:     **if**  $p^{⋆}(S)\leq \alpha /k$  **then**

8:       R←R∪{S}

9:     **end if**

10:     **if**  |R|>k  **then**

11:       k←k+1

12:       $R\leftarrow \{S\in R:p^{⋆}(S)\leq \alpha /k\}$

13:     **end if**

14:     **if**  ${\tilde{p}}^{⋆}(S)\leq \alpha /k$  **then**

15:       **for**  S′∈Children(S,S,NULL,∅)  **do**

16:         Enqueue(S′,Q)

17:       **end for**

18:     **end if**

19:   **end while**

20:   Solutions←∅

21:   **for**  S∈R  **do**

22:     **if**  p(S)≤α/k  **then**

23:       Add S to Solutions

24:     **end if**

25:   **end for**

26:   **return** Solutions

27: **end procedure**

Algorithm 2 explores C through a BFS traversal of the tree defined by the reduction P, exploiting Algorithm 1 (L.15) to invert the reduction and using this exploration to apply the Tarone testing procedure described in Section 2.2 (L7–12, 14), before finally testing the testable CCSs (L21-25). However, BFS is more memory intensive than depth-first search (DFS, see Section 4). In order to control the trade-off between speed and memory, we implemented a hybrid exploration scheme in which we allow each stage of the tree to be split into several batches. The tree is explored in BFS until some user-defined maximal width is reached at any level, at which point we start a DFS from the visited nodes. We then restart the exploration of the level in BFS.

### 3.5 Pruning more CCSs when controlling for an imbalanced categorical covariate

The envelope ${\tilde{p}}^{⋆}(S)=\min_{x\prime \geq x_{S}}p^{⋆}(S)$ introduced in Papaxanthos *et al.* (2016) verifies the monotonicity for any subgraph S because $S\prime ⊇S\Rightarrow x_{S\prime }\geq x_{S}$. However, the O(J log J) algorithm to compute this envelope only applies to the so called *potentially prunable* subgraphs which are such that $x_{S,j}\geq \max(n_{1,j},n_{2,j})$ for all subgroups j=1,…,J defined by the categorical covariate adjusted for by the CMH test. Pruning can therefore not be done from subgraphs for which at least one of the *J* groups has few occurrences of the corresponding covariate. This limitation arises in Lemma 2 of Papaxanthos *et al.* (2016), which characterizes the argmin of the envelope of a subgraph S. Lemma 4 lifts this restriction:

Lemma 4. *For any connected subgraph* S*, the envelope* ${\tilde{p}}^{⋆}$  *is attained for an optimum* $x_{S\prime }^{*}$  *such that* $x_{S\prime ,j}^{*}\in \{\max(x_{S,j},n_{1,j}),\max(x_{S,j},n_{2,j}),n_{j}\}$.

The proof is provided in Supplementary Section S-1.5. Lemma 4 exploits a cruder bound for groups that are not in the increasing regime of the minimal *p*-value. It recovers the Lemma 2 of Papaxanthos *et al.* (2016) for potentially prunable subgraphs, while offering an additional pruning opportunity for the other ones. If a subgraph was not potentially prunable only because it was missing the $x_{S,j}\geq \max(n_{1,j},n_{2,j})$ condition for one small group *j*, it may still be actually prunable since small groups of samples only affect the CMH test statistic marginally. On the other hand, if the condition is not verified for a large group or several small ones, the resulting envelope will be very loose and will not allow for pruning in practice. We provide some intuition in the Supplementary Appendix (Supplementary Fig. S3).

## 4 Experiments

We demonstrate the superiority of CALDERA in terms of computational speed, statistical power and biological interpretation. To do so, we rely on both simulated and real datasets.

### 4.1 Datasets and settings

To test the speed of the methods, we generate datasets with *n* samples represented by p∈[100:20,000] covariates, and a graph connecting these covariates. We vary the proportion *prop* of samples that are resistant, i.e. have a phenotype of 1, and the number of samples. We also perform exploration when changing the value of *α*, which impacts pruning. This leads to 4 scenarios to compare the runtimes of the methods, named **Speed 1** to **Speed 4**. More details on implementations and parameters can be found in Supplementary Section S-6. In order to test the speed gains provided by the new lower bound, we also explore an **Imbalance** in which we add a binary confounding variable, fixing *n *=* *100 and *p *=* *3000 but varying the balance of samples across the confounding variable.

To test the power of the different methods, we rely on a simulation where the ground truth is known, named **Exploration**. We generate a dataset with *n *=* *100 samples, 50 of each phenotype, where two genes A and B are present. Gene A is present for all samples, while gene B is only present for resistant samples. We introduce heterogeneity such that the DBG of the two genes is only linear at a coarse level (Fig. 1b). More details for the setting of those simulations are provided in Supplementary Section S-7.

We also rely on two real datasets, where we use compacted DBGs (de Bruijn, 1946; Pevzner *et al.*, 2001). In a DBG, *k*-mer are nodes and *k*-mer that overlap by *k – *1 nucleotides are connected by an edge. The graph is then compacted by reducing all linear sequences to a single node. The first dataset, which we name **Pseudomonas**, consists of the *n *=* *280 *P.aeruginosa* genomes along with their resistance phenotype to amikacin, used in DBGWAS (Jaillard *et al.*, 2018). The bacteria are partitioned based on k-mean into two distinct groups. The compacted DBG is constructed using the *k*-mers with *k *=* *31 (default) using DBGWAS, leading to a graph with over 2.3 million nodes and average degree ∼2.7. The second, named **Akkermansia**, consists of the *Akkermansia muciniphila* genomes collected in Karcher *et al.* (2021). We use host information as covariates: we want to identify genetic sequences that are associated with a body mass index over 30. The compacted DBG constructed over those *n *=* *401 strains has 1.3 million nodes with an average degree of ∼2.7. On these two real datasets, we rely on heuristics to choose the level *α* at which the FWER is controlled and the number of stages explored in the BFS search—a full exploration being too memory intensive. The level is fixed at the lowest value at which 10 CCSs at stage 1 of the BFS (i.e. unitig closures) are found significant. The stage is chosen by stopping when the number of unitigs covered by a significant CCS reaches a plateau—suggesting that further exploration would not bring much novelty.

### 4.2 Methods

On top of CALDERA, we use the following methods. COIN (Sese *et al.*, 2014) is to our knowledge the only described algorithm to identify significant CCSs, combining the enumeration method of COPINE with the LAMP algorithm. Minato *et al.* (2014) presented a provably superior version of LAMP, which we denote LAMP2. Since no implementation was provided in Sese *et al.* (2014), we implemented as a baseline COIN+LAMP2. Since CALDERA and COIN+LAMP2 both rely on the same statistical procedures (the identification of testable hypotheses with Fisher’s test), the set of significant CCSs found is the same regardless of the method. For this reason, we only use COIN+LAMP2 in the speed comparison, since the methods have the same power.

DBGWAS tests individual unitigs for association with a phenotype, using a linear mixed-model (LMM). We also benchmark three *k*-mer-based methods, available via the pyseer pipeline (Lees *et al.*, 2018), that recapitulates usual methods: a fixed-effect model without population effect, a LMM similar to the one used in DBGWAS, which is recommended, and an elastic-net model. Note that, since those methods, as well as DBGWAS, do not rely on graph exploration, they will not be benchmarked on the speed simulation portion, which solely focuses on that task.

### 4.3 Speed gains of CALDERA

In addition to COIN+LAMP2, we benchmark three versions of CALDERA. The first one, closest to COIN+LAMP2, is the DFS implementation. The second one is the BFS implementation, where we modify the enumeration order of the elements of C to promote pruning. The last is a parallelized BFS implementation, using five cores.

**Fig. 3.**
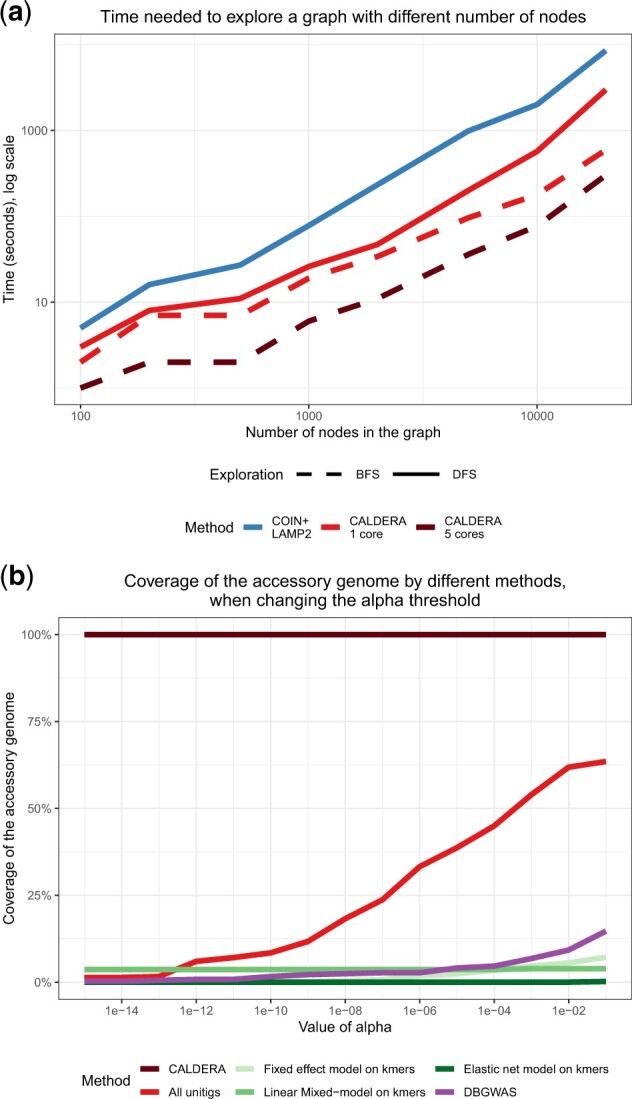
Results of CALDERA. (**a**) Run times for CALDERA and COIN+LAMP on graphs with various values of *p*. In this setting, *n *=* *100. (**b**) Proportion of all unitigs associated with the resistant phenotype that are found to be significant by CALDERA, the LAMP2 procedure on all unitigs and DBGWAS, as the value of *α* changes

*Benefit of CALDERA’s exploration scheme*: In Figure 3a, representing the results of **Speed 2**, we see that the ranking in speed is uniform over all value of *p*, with COIN+LAMP2 being the slowest, followed by the DFS and BFS implementation, and finally the parallelized version of CALDERA. For *p *=* *20 000, COIN+LAMP2 runs in 2h20 while the parallelized version of CALDERA takes 5 min. The ranking is the same for **Speed 1**, **Speed 3** and **Speed 4** (see Supplementary Section S-6). For example, for **Speed 1** and *p *=* *20 000, COIN+LAMP2 times out (2-day threshold) before finishing, while the parallelized version of CALDERA runs in 6 h. Over all parameter values, the average ratio of runtime for COIN+LAMP2 over CALDERA BFS with 5 cores is 76 and we tested CALDERA on graphs with up to *p *=* *100 000 nodes in 14 h. In terms of memory, CALDERA BFS and COIN+LAMP2 have near identical requirements, while the DFS implementation uses about 40% of the other methods. More details on memory usage can be found in Supplementary Section S-6.

On the larger **Pseudomonas** dataset, even CALDERA is unable to explore the entire C with the heuristic level $\alpha =10^{-6}$. We stop the exploration from any CCS that reaches a size of 2000 nucleotides or more (-Lmax 2000 option). We also observe that the unitigs covered by the significant CCSs reaches a plateau after the first six stages of the BFS. CALDERA took 3h20 on 4 cores (plus 2h30 to build the graph), using 200 Gb of RAM to complete these stages using batches of size 200 000, leading to $k_{0}=4,671,265$ potentially testable CCSs and only 39 significant ones. For comparison, after running for 24 h, COIN+LAMP2 was exploring the tree structure with a running $k_{0}=10^{5}$—i.e. had achieved about 2% of the exploration. DBGWAS runs in 2h45 (15 mn for the statistical test). *k*-mer-based methods benefit from a faster initial step of *k*-mer counting (1h30) but the statistical test is much longer, even using four cores: 2h30 for the fixed-effect and LMM, 3h30 for the elastic net method. Overall, CALDERA performs slower than DBGWAS but on par with the *k*-mer-based methods.

We provide a more general analysis of the computational cost of CALDERA against the number of BFS stages in Supplementary Section S-8 and recommend using a similar analysis and stop after a few stages in cases where a full exploration is no feasible.

*Benefit of CALDERA’s lower-bound on runtime for imbalanced population*: For extreme ratios—below 0.02—the new lower bound allows much more pruning and enumerates an order of magnitude fewer elements of C. Up to a ratio of 0.1, the new lower bound leads to a decrease of at least 10% in the number of explored subgraphs (see Supplementary Fig. S7).

### 4.4 Power gains of CALDERA

As mentioned above, COIN+LAMP2 and all versions of CALDERA rely on the same statistical procedures and therefore find an identical list of significant CCSs for a given level *α*. However, we can compare the power of CALDERA with DBGWAS and *k*-mer-based methods. We also use the LAMP2 procedure when testing All Unitigs separately, using Fisher’s test—like CALDERA.

We run all three methods on the dataset **Exploration** and measure how many of the 367 unitigs of gene B are called significant, when controlling the FWER at a varying level *α*. For CALDERA, a significant unitig is one that is contained in a significant CCS. Even when controlling the FWER at very low levels ($\alpha =10^{-16}$), CALDERA correctly recovers the entirety of the resistant gene. On the other hand, the other methods fail to ever recover the entire gene, even at α=0.1. This clearly show the enhanced power of CALDERA: because of variations along the genome, the association of any individual unitig with the phenotype is weak, while a covariate that jointly represents all 367 unitigs of the resistant gene is very strongly associated with that phenotype. The additional loss of power of *k*-mer methods stems from the larger multiple testing correction that they incur. Here, we also benchmarked Kover: although this method focuses on prediction and thus does not return *p*-values, its conjunction—disjunction approach could potentially identify the full gene as the best predictor of the phenotype. However, that is not the case and Kover only returns one *k*-mer as the best predictor.

We also apply those three methods to the **Pseudomonas** dataset. While there is no ground truth, this dataset contains two confirmed genetic variants linked to resistance to amikacin: a SNP on the aac(6′) gene, represented by one unitig, and the pHS87b plasmid, represented by 476 unitigs. This allows us to see how the methods handle those different scales. At the default $\alpha =10^{-6}$, CALDERA find the aac(6′) mutation as one CCS, and finds significant CCSs that covers 96% of the plasmid. Those two components represent 59% of all significant unitigs. In contrast, All Unitigs and DBGWAS do recover the mutation but only 34% and 0% of the plasmid, respectively. Even at α=0.1, All Unitigs and DBGWAS only recover respectively 72% and 8% of the plasmid. Moreover, while it is not possible to compute a false negative rate on a real dataset, we can see that, at this level, the two known sequences—the plasmid and the aac(6′) mutation—only represent 6% and 17% of all significant unitigs.

### 4.5 Simplified biological interpretation

Biological interpretation in DBGWAS or CALDERA happens at the component levels: significant unitigs or CCSs separated by only a few non-significant unitigs are displayed as one component. Unitigs can also be annotated using various databases, to enhance interpretation. Components are ranked in order of decreasing *p*-values, choosing the smallest *p*-value among all unitigs/CCSs. As such, both the number of components and their rankings will impact the ease of interpretation.

**Fig. 4.**
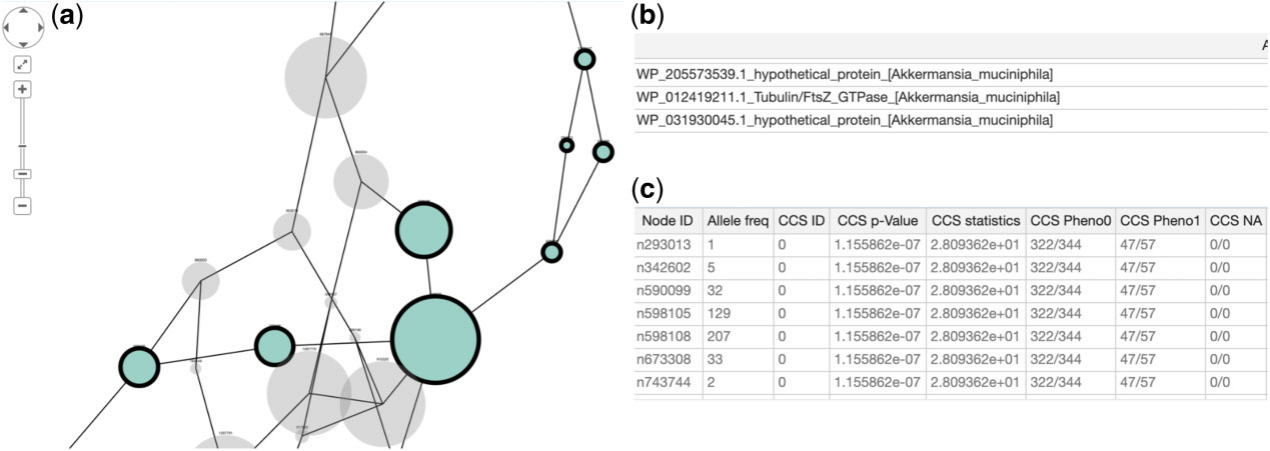
Akkermansia dataset: Tubulin/FtsZ_GTPase gene. Screenshot from the output of CALDERA. We select the first component, which is the one which contains the most significant CCS. (**a**) Unitigs belonging to the most significant CCS are colored in green (darker gray in the black and white version of the document). Other unitigs linked to the CCS in the DBG are colored in gray. Size denotes overall frequency, while a black contour denotes that the sequence of the unitig has a match in the database, here *RefSeq* (Tatusova *et al.*, 2016). (**b**) All significant hits on that database are listed in a panel, usually on top of the subgraph. (**c**) User can click on nodes to display information, or right-click to select all nodes from the same component. This contains info on the node, such as the frequency, the pattern of the associated CCS, or any match to the database (A color version of this figure appears in the online version of this article)

Figure 4 gives an example: we see the results of running CALDERA on the **Akkermansia** dataset. Only one CCS is significant, while DBGWAS returns no significant unitig. All the unitigs in the significant CCS (colored in green) are annotated, using the *RefSeq* database of all known *A.muciniphila* proteins as a reference (Tatusova *et al.*, 2016), and map to a common gene. This gene is not well annotated (hypothetical protein) but partially map to a Tubulin/FtsZ_GTPase *A.muciniphila* protein.

The plasmid returned as the first component of CALDERA on the **Pseudomonas** dataset can be seen in Supplementary Fig. S13. Visually, we can see clearly a broad circular graph, with local genetic variations. CALDERA returns eight components: the first is the entire plasmid, returned as one component. The second is the aac(6′) mutation. DBGWAS always ranks the aac(6′) mutation first but never returns the plasmid as one component, even when controlling the FWER at a level of 0.1 (three components, the first one ranked fourth). Moreover, at this level, DBWGAS returns 77 components, making the interpretation much harder.

## 5 Discussion

This article presented CALDERA, an algorithm to enumerate all significant CCSs. CALDERA is between one and two orders of magnitude faster than previously described exploration methods. It easily scales to large datasets, relying on an efficient structure on C and an exploration scheme that leverages the pruning opportunity offered by discrete statistics. This increased computational speed allows to deploy this method to DBG-based bacterial GWAS, which we demonstrate on two real examples. Moreover, we show that considering the CCSs, as done by CALDERA, leads to increased power and facilitates interpretation, compared to previous methods that perform statistical tests at the node level. CALDERA can better detect low signal caused by variability in genetic elements. It also returns larger and more coherent outputs that are easier to interpret.

We extensively discussed how CALDERA performs on bacterial GWAS. However, CALDERA can also be used for other tests of association involving a graph structure. We provide in Supplementary Section S-10 an example: we look at the association between SNPs on *Arabidopsis thaliana* genomes and a ‘date to flowering’ phenotype. In that setting, the graph is a regulatory network on the genes and the objective is to identify subnetworks whose disruption by at least one mutation is associated with the phenotype.

In settings where the node is a more natural object than the CCS, discrete testing can still be used to take advantage of Tarone (1990)’s procedure and increase power. However, pruning will no longer be possible, unless some other order can be established between nodes that preserve the order of minimal *p*-values.

For now, CALDERA does not scale to datasets with hundreds of millions of nodes that are possible in metagenome-wide association studies. Future work that focuses on incorporating pre-processing schemes before CALDERA would be needed to compact the graph to both reduce its size and facilitate pruning by increasing the average $\left|I(v_{j})\right|$.

## Supplementary Material

btac238_Supplementary_Data
