# Supplementary material for: CALDERA: finding all significant de Bruijn subgraphs for bacterial GWAS
Source: Bioinformatics. 2022 Jun 27;38(Suppl 1):i36–44. doi: 10.1093/bioinformatics/btac238 (PMC9235473; doi:10.1093/bioinformatics/btac238)
Supplement: btac238_Supplementary_Data [file btac238_supplementary_data.zip › btac238-suppl_data/ISBM_draft_simpler.pdf]

S-1 Proofs

S-1.1 Lemma 1: correctness of the closure

Lemma 1 provides that the operator cl is well defined on connected subgraphs.

Proof of Lemma 1 First let's show that there exists $\mathcal{S}' \in \mathcal{C}$ such that $\mathcal{I}(\mathcal{S}) = \mathcal{I}(\mathcal{S}')$ and $\mathcal{S} \subseteq \mathcal{S}'$. Let $\mathcal{S}'$ be a (inclusionwise) maximal connected subgraph containing $\mathcal{S}$ and such that $\mathcal{I}(\mathcal{S}) = \mathcal{I}(\mathcal{S}')$. By maximality of $\mathcal{S}'$, for every edge $(v_1, v_2) \in E$ with $v_1 \in \mathcal{S}'$ and $v_2 \notin \mathcal{S}'$, we have $\mathcal{I}(\mathcal{S}' \cup \{v_2\}) \neq \mathcal{I}(\mathcal{S}) = \mathcal{I}(\mathcal{S}')$, thus $\mathcal{S}' \in \mathcal{C}$.

Now let's show that such a subgraph is unique. Assume that there exits two different subgraphs $\mathcal{S}_1$ and $\mathcal{S}_2$ in $\mathcal{C}$ such that $\mathcal{S} \subseteq \mathcal{S}_1$ and $\mathcal{S} \subseteq \mathcal{S}_2$ with $\mathcal{I}(\mathcal{S}) = \mathcal{I}(\mathcal{S}_1) = \mathcal{I}(\mathcal{S}_2)$. Since $\mathcal{S}_1 \neq \mathcal{S}_2$, at least one of the subgraphs $\mathcal{S}_1 \setminus \mathcal{S}_2$ and $\mathcal{S}_2 \setminus \mathcal{S}_1$ is not empty. Assume without loss of generality that $\mathcal{S}_1 \setminus \mathcal{S}_2 \neq \emptyset$. Since $\mathcal{S}_1$ is connected and since $\mathcal{S}_1 \cap \mathcal{S}_2 \supseteq \mathcal{S} \neq \emptyset$, there is at least one edge (u, v) with $u \in \mathcal{S}_1 \cap \mathcal{S}_2$ and $v \in \mathcal{S}_1 \setminus \mathcal{S}_2$. This leads to a contradiction since the edge (u, v) is such that $u \in \mathcal{S}_2$, $v \notin \mathcal{S}_2$ and $\mathcal{I}(\mathcal{S}_2 \cup v) = \mathcal{I}(\mathcal{S}) = \mathcal{I}(\mathcal{S}_2)$, which is in contradiction with $\mathcal{S}_2 \in \mathcal{C}$.

S-1.2 Lemma 2: $\mathcal{P}$ is a valid reduction

Case if $\mathcal{I}(\mathcal{S}) = \mathcal{J}(\mathcal{S})$: Then, either $\mathcal{S} = \emptyset$ which has trivially no parent by this reduction. Or all nodes of $\mathcal{S}$ contain exactly the same pattern. For any $v \in \mathcal{S}$, $\mathcal{S} = cl(v)$. $\mathcal{S}$ is a root of our exploration. Its parent is $\emptyset \subseteq \mathcal{S}$. Note that, to avoid enumerating those roots more than once, we only start from $v_{\max} = \max \mathcal{S}$.

Case if $i_{\mathcal{S}}$ is defined: Then, $i_{\mathcal{S}} \in \mathcal{I}(\mathcal{S})$ so $\mathcal{S} \bigcap \mathbb{V}_{i_{\mathcal{S}}} \neq \emptyset$ and $i_{\mathcal{S}} \notin \mathcal{I}(\mathcal{S})$ so $\mathcal{S} \setminus \mathbb{V}_{i_{\mathcal{S}}} \neq \emptyset$. Therefore, there is at least one connected component in $\mathcal{S} \setminus \mathbb{V}_{i_{\mathcal{S}}}$. Moreover, any connected component of $\mathcal{S} \setminus \mathbb{V}_{i_{\mathcal{S}}}$ is included but not equal to $\mathcal{S}$. From (Haraguchi *et al.*, 2019), Lemma 1, we know that, if $\mathcal{S} \in \mathcal{C}$, any connected component of $\mathcal{S} \setminus \mathbb{V}_{i_{\mathcal{S}}}$ is also in $\mathcal{C}$. So any connected component of $\mathcal{S} \setminus \mathbb{V}_{i_{\mathcal{S}}}$ can be defined as a parent of $\mathcal{S}$. To identify a unique parent, we select $\mathcal{S}_p$, the one with the highest node number, $\mathcal{S}_p$. Since $\mathcal{S} \setminus \mathbb{V}_{i_{\mathcal{S}}} \neq \emptyset$ and $\mathcal{S}_p \subset \left(\mathcal{S} \bigcap \mathbb{V}_{i_{\mathcal{S}}}\right)$, then $\mathcal{S}_p \subsetneq \mathcal{S}$: it is a strictly smaller subgraph by inclusion. This proves that reduction defines a unique parent and proves that the reduction is valid.

S-1.3 Lemma 3: conditions (C1-3) are necessary and sufficient for $\mathcal{S} = \mathcal{P}(\mathcal{S}')$

We first prove the following lemma:

Lemma 5. *For two subgraphs $\mathcal{S}_1, \mathcal{S}_2 \in \mathcal{C}$, if $\mathcal{S}_1 \subset \mathcal{S}_2$, then $cl(\mathcal{S}_1) \subset cl(\mathcal{S}_2)$.*

Proof: Since $\mathcal{S}_1 \subset cl(\mathcal{S}_1)$ and $\mathcal{S}_1 \subset \mathcal{S}_2 \subset cl(\mathcal{S}_2)$, we then known that $cl(\mathcal{S}_1) \bigcap cl(\mathcal{S}_2) \neq \emptyset$. Let' now assume that $cl(\mathcal{S}_1) \not\subseteq cl(\mathcal{S}_2)$. Since $cl(\mathcal{S}_1)$ is a connected subgraph, there exists $v \in cl(\mathcal{S}_1) \bigcap Ne(cl(\mathcal{S}_2))$. $v \in cl(\mathcal{S}_1)$ so $\mathcal{I}(v) \subset \mathcal{I}(cl(\mathcal{S}_1)) = \mathcal{I}(\mathcal{S}_1)$. But $\mathcal{S}_1 \subset \mathcal{S}_2$ so $\mathcal{I}(\mathcal{S}_1)] \subset \mathcal{I}(\mathcal{S}_2)$. Therefore, $\mathcal{I}(v) \subset \mathcal{I}(cl(\mathcal{S}_2))$ and $v \in Ne(cl(\mathcal{S}_2))$. This contradicts the definition of the closure. So we prove the lemma.

S-1.3.1 Proof that for any $\mathcal{S}'$, $(\mathcal{S} = \mathcal{P}(\mathcal{S}'), \mathcal{S}')$ verify $(C1-3)$

$\mathcal{S} \subset \mathcal{S}' \setminus \mathbb{V}_{i_{\mathcal{S}'}}$ so $i_{\mathcal{S}'} \notin \mathcal{I}(\mathcal{S})$. This proves (1). $\max\{v' \in \mathcal{S}' \setminus \mathbb{V}_{i_{\mathcal{S}'}}\} \in \mathcal{S}$ by construction of the parent so $\max\{v' \in \mathcal{S}' \setminus \mathbb{V}_{i_{\mathcal{S}'}}\} \leq \max \mathcal{S}$. Moreover, $\mathcal{S} \subset \mathcal{S}' \setminus \mathbb{V}_{i_{\mathcal{S}'}}$ so $\max \mathcal{S} \leq \max\{v' \in \mathcal{S}' \setminus \mathbb{V}_{i_{\mathcal{S}'}}\}$. So $\max\{v' \in \mathcal{S}' \setminus \mathbb{V}_{i_{\mathcal{S}'}}\} = \max \mathcal{S}$, this proves (2).

Suppose (3) is false. Then, there exists $v \in Ne(\mathcal{S}) \bigcap (\mathcal{S}' \setminus \mathbb{V}_{i_{\mathcal{S}'}})$. $\mathcal{S} \bigcup \{v\} \supset \mathcal{S}' = cl(\mathcal{S})'$ so $\mathcal{S}_2 = cl(\mathcal{S} \bigcup \{v\}) \subset \mathcal{S}'$, $\max \mathcal{S}_2 = \max\{v' \in \mathcal{S}' \setminus \mathbb{V}_{i_{\mathcal{S}'}}\}$ since $\mathcal{S} \subset \mathcal{S}_2$ and $\mathcal{S}_2 \bigcap \mathbb{V}_{i_{\mathcal{S}'}} = \emptyset$ so $\mathcal{S}_2 \subset \mathcal{P}(\mathcal{S}')$. But $\mathcal{S}2 \supsetneq \mathcal{S} = \mathcal{P}(\mathcal{S})$. This is not possible. So (3) is true.

This proves the implication in the first sense.

S-1.3.2 Proof that for any $(\mathcal{S}, \mathcal{S}')$ that verify $(C1-3)$, $\mathcal{S} = \mathcal{P}(\mathcal{S}')$

We consider two closed connected subgraph $\mathcal{S}, \mathcal{S}' \in \mathcal{C}$ that verify (1-3). We want to prove that $\mathcal{P}(\mathcal{S}') = \mathcal{S}$. Point (1) insures that $\mathcal{S} \subsetneq (\mathcal{S}' \setminus \mathbb{V}_{i_{\mathcal{S}'}})$. Since $\mathcal{S} \in \mathcal{C}$ and contains the maximal node (from (2)), this ensures that $\mathcal{S} \subseteq \mathcal{P}(\mathcal{S}')$.

Suppose $\mathcal{S} \subsetneq \mathcal{P}(\mathcal{S}')$. Then, $\mathcal{P}(\mathcal{S}') \setminus \mathcal{S} \neq \emptyset$. In particular, since $\mathcal{S}$ and $\mathcal{P}(\mathcal{S}')$ are both connected subgraphs, there exists $v' \in \left(\mathcal{P}(\mathcal{S}') \setminus \mathcal{S}\right) \bigcap Ne(\mathcal{S})$. Since this neighbour is in $\mathcal{P}(\mathcal{S}')$, it is also in $\mathcal{S}' \setminus \mathbb{V}_{i_{\mathcal{S}'}}\}$. That is impossible from (3). So $\mathcal{S} = \mathcal{P}(\mathcal{S}')$. (Note that point (3) includes the fact that $i_{\mathcal{S}'} \in \mathcal{I}(v)$).

This proves the converse implication.

S-1.4 Theorem 1: Algorithm 1 correctly inverts the reduction

We consider a subgraph $\mathcal{S}' \in \mathcal{S}$ and its parent $\mathcal{S} = \mathcal{P}(\mathcal{S}')$.

We first show two lemmas

Lemma 6. *For two subgraphs $\mathcal{S}_1, \mathcal{S}_2 \in \mathcal{C}$, if $\mathcal{S}_1 \subset \mathcal{S}_2$, then $i_{\mathcal{S}_1} \leq i_{\mathcal{S}_2}$.*

Proof:

$$\begin{aligned}
\mathcal{S}_1 \subset \mathcal{S}_2 &\implies \mathcal{I}(\mathcal{S}_1) \subset \mathcal{I}(\mathcal{S}_2) && \text{and} \quad (1)\\
\mathcal{S}_1 \subset \mathcal{S}_2 &\implies \mathcal{J}(\mathcal{S}_2) \subset \mathcal{J}(\mathcal{S}_1) && (2)\\
(1) \text{ and } (2) &\implies (\mathcal{I}(\mathcal{S}_1) \setminus \mathcal{J}(\mathcal{S}_1)) \subset (\mathcal{I}(\mathcal{S}_2) \setminus \mathcal{J}(\mathcal{S}_2)) && (3)\\
&\implies i_{\mathcal{S}_1} \leq i_{\mathcal{S}_2} && (4)
\end{aligned}$$

Lemma 7. *For a subgraph $\mathcal{S}' \in \mathcal{C}$ such that $\mathcal{S} = \mathcal{P}(\mathcal{S}') \neq \emptyset$, any subgraph $\mathcal{S}_2 \in \mathcal{C}$ that verifies:*

- $\mathcal{S} \subsetneq \mathcal{S}_2$
- $\mathcal{S}_2 \subset \mathcal{S}'$

is a child of $\mathcal{S}$, that is $\mathcal{P}(\mathcal{S}_2) = \mathcal{S}$

Proof: We know that $\mathcal{S} \subsetneq \mathcal{S}_2$ so $Ne(\mathcal{S}) \bigcap \mathcal{S}_2 \neq \emptyset$. Since $\mathcal{S}_2 \subset \mathcal{S}'$, $Ne(\mathcal{S}) \bigcap \mathcal{S}_2 \subset \mathcal{S}'$ so, from (3) for $\mathcal{S}, \mathcal{S}'$, we have $Ne(\mathcal{S}) \bigcap \mathcal{S}_2 \subset \mathbb{V}_{i_{\mathcal{S}'}}$. So $i_{\mathcal{S}'} \in \mathcal{I}(\mathcal{S}_2)$. $i_{\mathcal{S}'} \notin \mathcal{I}(\mathcal{S})$ so $i_{\mathcal{S}'} \notin \mathcal{J}(\mathcal{S}_2)$. Therefore, $i_{\mathcal{S}'} \leq i_{\mathcal{S}_2}$. But since $\mathcal{S}_2 \subset \mathcal{S}'$, $i_{\mathcal{S}'} \geq i_{\mathcal{S}_2}$. So $i_{\mathcal{S}'} = i_{\mathcal{S}_2}$. Then, we know that $\mathcal{S}, \mathcal{S}_2$ verifies (1). Since $\mathcal{S} \subsetneq \mathcal{S}_2$, we also have (2). Finally $\{v' \in \mathcal{S}_2 \setminus \mathbb{V}_{i_{\mathcal{S}_2}} : v' \in Ne(\mathcal{S})\} = \{v' \in \mathcal{S}_2 \setminus \mathbb{V}_{i_{\mathcal{S}'}} : v' \in Ne(\mathcal{S})\} \subset \{v' \in \mathcal{S}' \setminus \mathbb{V}_{i_{\mathcal{S}'}} : v' \in Ne(\mathcal{S})\} = \emptyset$. This proves (3). Since we have (1-3), we know that $\mathcal{P}(\mathcal{S}_2) = \mathcal{S}$.

Main proof: Now let us prove the main result: Let' consider a subgraph $\mathcal{S}$ and assume that we cannot generate $\mathcal{S}'$ that verifies $\mathcal{P}(\mathcal{S}') = \mathcal{S}$ with the procedure from algorithm 1.

We take $v \in Ne(\mathcal{S}) \bigcap \mathbb{V}_{i'_{\mathcal{S}}}$. $\mathcal{S}_d = cl(\mathcal{S} \bigcup \{v\})$ verifies $\mathcal{S}_d \in \mathcal{C}$, $\mathcal{S} \subseteq \mathcal{S}_d$ and $\mathcal{S}_d \subset \mathcal{S}'$ (from Lemma 5). So, from lemma 7, we know that $\mathcal{P}(\mathcal{S}_d) = \mathcal{S}$ so $(\mathcal{S}, \mathcal{S}_d)$ verifies (1-3). Therefore, we generate $\mathcal{S}_d$ through lines 4-8 of algorithm 1. (Note that we can call $\mathcal{S}_d$ a *direct* child of $\mathcal{S}$, and the other subgraphs generated via lines 11-15 as its siblings).

We know that we generate through algorithm 1 from $\mathcal{S}_d$ a set of subgraphs $\mathcal{S}_s \subset \mathcal{S}'$. Let's then consider the largest $\mathcal{S}'' \subsetneq \mathcal{S}'$ generated with the algorithm 1, that is the one with the largest number of nodes. We note $\mathcal{T}$ the itemtable that accompanies the creation of $\mathcal{S}''$. Note that we know that $i_{\mathcal{S}''} = i_{\mathcal{S}'}$ since $i_{\mathcal{S}''} \leq i_{\mathcal{S}'}$ (lemma 6) and $i_{\mathcal{S}''} \geq i_{\mathcal{S}_d} = i_{\mathcal{S}'}$.

By assumption, $\mathcal{S}'' \subsetneq \mathcal{S}'$. Therefore, there exists a neighbour $v \in Ne(\mathcal{S}'') \bigcap \mathcal{S}'$ since $\mathcal{S}'$ and $\mathcal{S}''$ are connected subgraphs. Moreover, $\mathcal{S}_2 = cl(\mathcal{S}'' \bigcup \{v\}))$ is a child of $\mathcal{S}$ by Lemma 7 so $(\mathcal{S},$ verify (1-3) by Lemma 2.

Case 1: $\{\mathcal{I} \in \mathcal{T} : \mathcal{I} \subset \mathcal{I}(\mathcal{S}_2))\} = \emptyset$*:* Since $\mathcal{S}_2 \subset \mathcal{S}'$, $i_{\mathcal{S}_2} \leq i_{\mathcal{S}'}$. However, since $\mathcal{S}'' \subset \mathcal{S}_2$, $i_{\mathcal{S}_2} \geq i_{\mathcal{S}''} = i_{\mathcal{S}'}$. So $i_{\mathcal{S}_2} = i_{\mathcal{S}'}$. Moreover, we already know that $(\mathcal{S}, \mathcal{S}_2)$ verify (1-3). We therefore try $\mathcal{S}_2$ following Line 5, we checked all the conditions (Line 11-12). That means we can created $\mathcal{S}_2 \supsetneq \mathcal{S}''$ which contradicts our assumption that $\mathcal{S}''$ is the largest closed subgraph strictly included in $\mathcal{S}'$ that could be generated.

Case 2: $\{\mathcal{I} \in \mathcal{T} : \mathcal{I} \subset \mathcal{I}(\mathcal{S}_2))\} \neq \emptyset$*:* We note $v_1, \ldots, v_l$ the sequence that created $\mathcal{S}''$ from $\mathcal{S}_d$ through successive additions and closures (following Line 5). At one point in that process, we added a pattern to $\mathcal{T}$ that is now contained in $\mathcal{I}(cl(\mathcal{S}'' \bigcup \{v\}))$, let's say when adding v_k. Note that it cannot be the same pattern otherwise $\mathcal{S}''$ would not be closed. This pattern was linked to another equivalence group (Line 13).. If we consider v' a node from that group, we will then construct a subgraph using the sequence $v_1, \ldots, v_{k-1}, v', v_k, \ldots, ...v_l$. Note that since at each new addition, the constructed graph is included in $\mathcal{S}_2$, it's also included in $\mathcal{S}'$. Moreover, each one contains $\mathcal{S}$ and a node from $\mathbb{V}_{i_{\mathcal{S}'}}$ by construction (since it contains $\mathcal{S}_d$). So, using Lemma 2, we know that those additions verifies the conditions of Lines 11 and 12: they are valid additions according to our algorithm. This way, we can create a subgraph that contains $\mathcal{S}''$ and v' with our procedure. This contradicts our assumption that $\mathcal{S}''$ is the largest closed subgraph strictly included in $\mathcal{S}'$ that could be generated.

This proves that all $\mathcal{S}'$ will be generated from $\mathcal{S}$ and therefore that we have properly inverted the reduction

S-1.5 Proof of Lemma 4

The next two lemmas are directly taken from (Papaxanthos *et al.*, 2016).

Minimal p-value of the CMH test

Lemma 8 ((Papaxanthos *et al.*, 2016)). *The minimal p-value of the CMH test can be computed in* $O(J)$.

Proof The p-value associated with the CHM test, conditioning on the margins of all the tables, is:

$$\begin{aligned} p_{CMH}(\mathcal{S}, \mathbf{Y}, \mathbf{C}) =& 1 - \mathbf{F}_{\chi^2_1}\Big(\frac{\big(\sum_{j=1}^J a_{\mathcal{S},j} - \frac{x_{\mathcal{S},j} n_{1,j}}{n_j}\big)^2}{\sum_{j=1}^J \frac{n_{1,j}}{n_j - 1}\frac{n_{2,j}}{n_j} x_{\mathcal{S},j}(1 - \frac{x_{\mathcal{S},j}}{n_j})}\Big) \\ =& 1 - \mathbf{F}_{\chi^2_1}\Big(\frac{\big(a_{\mathcal{S}} - \sum_{j=1}^J \frac{x_{\mathcal{S},j} n_{1,j}}{n_j}\big)^2}{\sum_{j=1}^J \frac{n_{1,j}}{n_j - 1}\frac{n_{2,j}}{n_j} x_{\mathcal{S},j}(1 - \frac{x_{\mathcal{S},j}}{n_j})}\Big) \\ =& 1 - \mathbf{F}_{\chi^2_1}\Big(T_{\mathcal{S}}(a_{\mathcal{S}}, x_{\mathcal{S}})\Big) \end{aligned}$$

Since $\mathbf{F}_{\chi^2_1}$ is monotonically increasing, the minimal p-value is obtained for the smallest value $T_{\mathcal{S}}(a_{\mathcal{S}}, x_{\mathcal{S}}$. This is a function of $a_{\mathcal{S}}$ that is quadratic with a positive definite hessian(Papaxanthos *et al.*, 2016) so the function is maximal for $\min a_{\mathcal{S}}$ or $\max a_{\mathcal{S}}$. We have that $a_{\mathcal{S},j,\min} = 0$ if $x_{\mathcal{S},j} \leq n_{2,j}$ and $a_{\mathcal{S},j,\min} = x_{\mathcal{S},j} - n_{2,j}$; and $a_{\mathcal{S},j,\max} = x_{\mathcal{S},j}$ for $x_{\mathcal{S},j} \leq n_{1,j}$ and $a_{\mathcal{S},j,\max} = n_{1,j}$ otherwise. So, $T^{\max}_{\mathcal{S}}(x_{\mathcal{S}}) = \max(T^l_{\mathcal{S}}, T^r_{\mathcal{S}})$ where

$$\begin{aligned} T^l_{\mathcal{S}} =& \frac{\big(\sum_{j=1}^J a_{\mathcal{S},j,\min} - \frac{x_{\mathcal{S},j} n_{1,j}}{n_j}\big)^2}{\sum_{j=1}^J \frac{n_{1,j}}{n_j - 1}\frac{n_{2,j}}{n_j} x_{\mathcal{S},j}(1 - \frac{x_{\mathcal{S},j}}{n_j})} \\ T^r_{\mathcal{S}} =& \frac{\big(\sum_{j=1}^J a_{\mathcal{S},j,\max} - \frac{x_{\mathcal{S},j} n_{1,j}}{n_j}\big)^2}{\sum_{j=1}^J \frac{n_{1,j}}{n_j - 1}\frac{n_{2,j}}{n_j} x_{\mathcal{S},j}(1 - \frac{x_{\mathcal{S},j}}{n_j})} \end{aligned}$$

Computing the envelope

Definition 3. *For each* $\mathcal{S} \in \mathcal{C}$*, the envelope of* $\mathcal{S}$ *is defined as* $\tilde{p}^\star(\mathcal{S}) \equiv \min_{\mathcal{S}'':x_{\mathcal{S}''} \geq x_{\mathcal{S}}} p^*(\mathcal{S}'')$.

Lemma 9 ((Papaxanthos *et al.*, 2016)). *If a subgraph is prunable, i.e* $\tilde{p}^\star(\mathcal{S}) > \alpha/k$*, then any subgraph* $\mathcal{S}' \supset \mathcal{S}$ *is also prunable*

Proof :

$$\begin{aligned} \mathcal{S}' \supset \mathcal{S} &\implies \{\mathcal{S}'' : x_{\mathcal{S}''} \geq x_{\mathcal{S}}\} \supset \{\mathcal{S}'' : x_{\mathcal{S}''} \geq x'_{\mathcal{S}}\} \\ &\implies \min_{\mathcal{S}'':x_{\mathcal{S}''} \geq x_{\mathcal{S}}} p^*(\mathcal{S}'') \leq \min_{\mathcal{S}'':x_{\mathcal{S}''} \geq x'_{\mathcal{S}}} p^*(\mathcal{S}'') \end{aligned}$$

$\tilde{p}^\star(\mathcal{S}) > \alpha/k$ and above $\implies \tilde{p}^\star(\mathcal{S}') > \alpha/k$

Proof of Lemma 4 We consider the function

$$T_l(x'_{\mathcal{S}}) = \frac{\big(a_{\mathcal{S}',\min} - \sum_{j=1}^J \frac{x_{\mathcal{S}',j} n_{1,j}}{n_j})\big)^2}{\sum_{j=1}^J \frac{n_{1,j}}{n_j - 1}\frac{n_{2,j}}{n_j} x_{\mathcal{S}',j}(1 - \frac{x_{\mathcal{S}',j}}{n_j})}$$

with $x_{\mathcal{S},j} \leq x_{\mathcal{S}',j} \leq n_j$ and we will look at the partial derivatives. We add a few notations:

$$\begin{aligned} \mu =& \frac{n_{1,j}}{n_j - 1}\frac{n_{2,j}}{n_j} \\ K =& \sum_{j=1}^J \frac{n_{1,j}}{n_j - 1}\frac{n_{2,j}}{n_j} x_{\mathcal{S}',j}(1 - \frac{x_{\mathcal{S}',j}}{n_j}) \\ K_{-i} =& \sum_{j=1, j\neq i}^J \frac{n_{1,j}}{n_j - 1}\frac{n_{2,j}}{n_j} x_{\mathcal{S}',j}(1 - \frac{x_{\mathcal{S}',j}}{n_j}) \\ S =& \sum_{j=1}^J \frac{x_{\mathcal{S}',j} n_{1,j}}{n_j} - a_{\mathcal{S},j,\min} \\ S_{-i} =& \sum_{j=1, j\neq i}^J \frac{x_{\mathcal{S}',j} n_{1,j}}{n_j} - a_{\mathcal{S},j,\min} \\ a'_{\mathcal{S},\min} =& \frac{\partial a_{\mathcal{S},\min}}{\partial x_{\mathcal{S}',i}} \end{aligned}$$

We then have

$$\begin{aligned} \frac{\partial T_l(x_{\mathcal{S}'})}{\partial x_{\mathcal{S}',i}} =& N_l(x_{\mathcal{S}'}) \times D_l(x_{\mathcal{S}'}) && \text{where} \\ N_l(x_{\mathcal{S}'}) =& 2(\frac{n_{1,i}}{n_i} - a'_{\mathcal{S},\min})K - (1 - 2\frac{x_{\mathcal{S}',i}}{n_i})\mu S && \text{and} \\ D_l(x_{\mathcal{S}'}) =& \frac{S}{K^2} \end{aligned}$$

For all j, $\frac{x_{\mathcal{S}',j} n_{1,j}}{n_j} - a_{\mathcal{S},j,\min} \geq \frac{x_{\mathcal{S}',j} n_{1,j}}{n_j} - x_{\mathcal{S}',j} + n_{2,j} = n_{2,j}(1 - \frac{x_{\mathcal{S}',j}}{n_j}) \geq 0$ so $D_l(x_{\mathcal{S}'}) \geq 0$. We only need look at $N_l(x_{\mathcal{S}'})$ to find maxima. We can also note that consequently, $S \geq 0$ and $S_{-i} \geq 0$.

$$
\begin{aligned}
N_l(x_{\mathcal{S}'}) =& 2(\frac{n_{1,i}}{n_j} - a'_{\mathcal{S},\min})K - (1 - 2\frac{x_{\mathcal{S}',i}}{n_j})\mu S \\
=& 2\frac{n_{1,i}}{n_i}K_{-i} + 2\frac{n_{1,i}}{n_i}\mu x_{\mathcal{S}',i}(1 - \frac{x_{\mathcal{S}',i}}{n_i}) - \\
& 2a'_{\mathcal{S},\min}K_{-i} - 2a'_{\mathcal{S},\min}\mu x_{\mathcal{S}',i}(1 - \frac{x_{\mathcal{S}',i}}{n_i}) - \\
& \mu S_{-i} - \mu(\frac{x_{\mathcal{S}',i}n_{1,i}}{n_i} - a_{\mathcal{S},i,\min}) + 2\frac{x_{\mathcal{S}',i}}{n_i}\mu S_{-i} + \\
& 2\frac{x_{\mathcal{S}',i}}{n_i}\mu(\frac{x_{\mathcal{S}',i}xn_{1,i}}{n_i} - a_{\mathcal{S},i,\min}) \\
=& [2\frac{n_{1,i}}{n_i}K_{-i} - 2a'_{\mathcal{S},\min}K_{-i} - \mu S_{-i}] + \\
& x_{\mathcal{S}',i}[2\mu\frac{n_{1,i}}{n_i} - 2a'_{\mathcal{S},\min}\mu - \mu\frac{n_{1,i}}{n_i} + 2\frac{\mu}{n_i}S_{-i}] + \\
& - 2\frac{n_{1,i}}{n_i^2}\mu x^2_{\mathcal{S}',i} + 2\frac{n_{1,i}}{n_i^2}\mu x^2_{\mathcal{S}',i} + 2a'_{\mathcal{S},\min}\mu\frac{x^2_{\mathcal{S}',i}}{n_i} - \\
& 2a_{\mathcal{S},i,\min}\mu\frac{x_{\mathcal{S}',i}}{n_i} + \mu a_{\mathcal{S},\min}
\end{aligned}
$$

We then have two cases:

1. $x_{\mathcal{S}',i} \leq n_{2,i}$: Then, $a_{\mathcal{S},i,\min} = 0$ and $a'_{\mathcal{S},\min} = 0$. So:

$$
N_l(x_{\mathcal{S}'}) = [2\frac{n_{1,i}}{n_i}K_{-i} - \mu S_{-i}] + x_{\mathcal{S}',i}[\mu\frac{n_{1,i}}{n_i} + 2\frac{\mu}{n_i}S_{-i}]
$$

   It is an affine function with a positive slope. Moreover, at the boundary at $n_{2,i}$, the function is positive. So the function is maximal at $n_{2,i}$.

2. $x_{\mathcal{S}',i} \geq n_{2,i}$: Then, $a_{\mathcal{S},i,\min} = x_{\mathcal{S}',i} - n_{2,i}$ and $a'_{\mathcal{S},\min} = 1$. So:

$$
\begin{aligned}
N_l(x_{\mathcal{S}'}) =& [2\frac{n_{1,i}}{n_i}K_{-i} - 2K_{-i} - \mu S_{-i} - \mu n_{2,i}] + \\
& x_{\mathcal{S}',i}[\mu\frac{n_{1,i}}{n_i} - 2\mu + \mu + 2\mu\frac{n_{1,i}}{n_i} + 2\frac{\mu}{n_i}S_{-i}] + \\
& 2\mu\frac{x^2_{\mathcal{S}',i}}{n_i} - 2\mu\frac{x^2_{\mathcal{S}',i}}{n_i} \\
=& [2\frac{n_{1,i}}{n_i}K_{-i} - 2K_{-i} - \mu S_{-i}] + \\
& x_{\mathcal{S}',i}[\mu\frac{n_{2,i}}{n_i} + 2\frac{\mu}{n_i}S_{-i}]
\end{aligned}
$$

   So $N_l(x_{\mathcal{S}'})$ is an affine by-piece function of $x_{\mathcal{S}',i}$, whose slope $A_l(x_{\mathcal{S}',-i}) \geq 0$. So, the only possible maxima are at the boundary, where $x_{\mathcal{S}',i} = n_{2,i}$ or $x_{\mathcal{S}',i} = n_i$. Since this is true for all values of $x_{\mathcal{S}',-i}$, we know that we can only achieve a maximum for T_l at the boundaries. So the only two possible maxima are $n_{2,i}$ and n_i. Note that, in the case where $x_{\mathcal{S}',i} \geq n_{2,i}$, then the possible maxima becomes $x_{\mathcal{S}',i}$ and n_i so in general, the two possible maxima for $T_l(x'_{\mathcal{S}})$ are $\max\{n_{2,i}, x_{\mathcal{S}',i}\}$ and n_i.

The same proof holds for T_r (given the symmetry of the expressions), where the maxima is in $\{\max(x_{\mathcal{S},i}, n_{1,i}), n_i\}$. This proves the lemma.We have reduced the space of possibilities from $O(m^J)$ to $O(2^J)$ (with m the geometric mean of $x_{\mathcal{S}'}$).

We now need to show how to compute this value in $O(J\log(J))$. For this, we rely on the following theorem.

Lemma 10 ((Papaxanthos *et al.*, 2016)). *Let $\mathcal{S}$ be a potentially testable subgraph and define $\beta^l_{\mathcal{S}',1} = \frac{n_{1,j}x_{\mathcal{S}',j}}{n_j^2}$ and $\beta^l_{\mathcal{S}',1} = \frac{n_{2,j}x_{\mathcal{S}',j}}{n_j^2}$, for $j \in \{1,\ldots,J\}$ Let π_l and π_r be permutations of $\{1,\ldots,J\}$ such that $\beta^l_{\mathcal{S}',\pi_l(1)} \leq \cdots \leq \beta^l_{\mathcal{S}',\pi_l(J)}$ and $\beta^r_{\mathcal{S}',\pi_r(1)} \leq \cdots \leq \beta^r_{\mathcal{S}',\pi_r(J)}$, respectively.*
Then, there exist $\kappa \in \{1,\ldots,J\}$ such that the optimum $x^*_{\mathcal{S}'}$ satisfies either

- *$x^*_{\mathcal{S}',\pi_l(j)} = x_{\mathcal{S},\pi_l(j)}$ for $j \leq \kappa$ and $x^*_{\mathcal{S}',\pi_l(j)} = n_j$ otherwise*

or

- *$x^*_{\mathcal{S}',\pi_r(j)} = x_{\mathcal{S},\pi_r(j)}$ for $j \leq \kappa$ and $x^*_{\mathcal{S}',\pi_r(j)} = n_j$ otherwise*

Proof of lemma 10. We will do the proof for $T_l(x'_{\mathcal{S}})$. The proof for $T_r(x'_{\mathcal{S}})$ is identical, up to notation.

We can note that since the optimal is at least equal to $n_{2,j}$, the value of $a_{\mathcal{S},j,\min}$ is $x_{\mathcal{S}',j} - n_{2,j}$. We note $\beta_j = \frac{n_{1,j}x^*_{\mathcal{S}',j}}{n_j^2}$. We also note $l(\beta_j) = n_{1,j}n_j(1 - \frac{n_j\beta_j}{n_{1,j}}) = n_{2,j}(1 - \frac{x^*_{\mathcal{S}',j}}{n_j})$. With those notations, we can write

$$
T_l(x'_{\mathcal{S}}) = \frac{\sum_{j=1}^J l(\beta_j)}{\sum_{j=1}^J \beta_j l(\beta_j)}
$$

It is straightforward to see that, if $x^*_{\mathcal{S}',j} = n_j$, then $l(\beta_j) = 0$. We are then exactly in the setting of Papaxanthos *et al.* (2016) and we refer the reader to the proof in the supplementary, p.3-6.

This shows that we can compute the envelope in $J\log(J)$

S-2 Efficient implementation of `CALDERA`

We consider a subgraph $\mathcal{S}'$ created following Algorithm 1, in the second case (Lines 11-15). We therefore have $\mathcal{S}, \mathcal{S}_p$ such that: $\mathcal{P}(\mathcal{S}') = \mathcal{P}(\mathcal{S}) = \mathcal{S}_p$ and $i_{\mathcal{S}'} = i_{\mathcal{S}}$. After creating $\mathcal{S}'$, we explore its children, with an itemtable $\mathcal{T}$. All elements of $\texttt{Children}(\mathcal{S}', \mathcal{S}_p, i_{\mathcal{S}'}, \mathcal{T})$ will have a pattern which includes $\mathcal{I}(\mathcal{S}')$. Moreover, by definition of the equivalence groups, we already know that $\{\mathcal{I} \in \mathcal{T} : \mathcal{I} \subset \mathcal{I}(\mathcal{S}')\} = \emptyset$. Therefore, when constructing $\mathcal{S}'' \in \texttt{Children}(\mathcal{S}', \mathcal{S}_p, i_{\mathcal{S}'}, \mathcal{T})$, only the elements in $\mathcal{I}(\mathcal{S}'') \setminus \mathcal{I}(\mathcal{S}')$ need to be considered.

We store $\mathcal{T}$ as a matrix of binary patterns. Therefore, some columns can be deleted without loss of information: in Line 13 of algorithm 1, we only keep the columns that are not in $\mathcal{I}(\mathcal{S})$. As the `Children` function is called recursively, the itemtable $\mathcal{T}$ will grow in the number of patterns saved (*i.e* number of rows) but the memory footprint of each pattern will be smaller (*i.e* fewer columns).

S-3 Background on the minimal p-value

S-4 Benefits of the new envelope

To demonstrate the situations where the new envelope is beneficial, and where it is not, we consider a situation where $n = 280$, $J = 2$. Then, we look at two cases: $n_1 = n_2 = 140$ and $n_1 = 13 \times n_2 = 260$. In both settings, we compute the envelope as defined in Papaxanthos *et al.* (2016) and in our case. Then, for $\alpha = 10^{-8}$ (a value that we used in practice) and for all possible values of $\{x_{\mathcal{S},1}, x_{\mathcal{S},2}\}$, we consider whether we would prune (for $k = 1$) using the definition of the envelope from Papaxanthos *et al.* (2016) or the extended new bound defined in this paper. The new bound nearly doubles the space of prunable subgraphs when there is a clear imbalance, as evidenced in Fig S3b, while it has no effect when the two populations are perfectly balanced, as in Fig S3a.

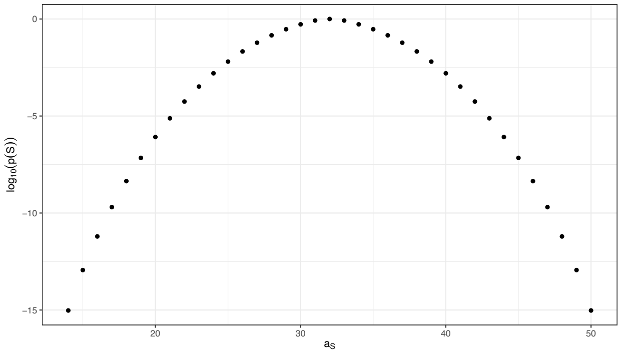

Fig. S1: *Finite numbers of possible p-values (log scale) for a fixed value of $n1 = 50$ and $x_{\mathcal{S}} = 64$. Using the notation from table 1, with $J = 1$, $n_1 = 50$, $n = 100$ and $x_{\mathcal{S}} = 64$, the p-value of the χ^2 test is computed for all possible values of $a_{\mathcal{S}}$. Since there are only a finite number of possible $a_{\mathcal{S}}$ values, there are a finite number of possible p-values, and therefore a smallest one. This minimal p-value can be computed from $x_{\mathcal{S}}$, $n1$ and n alone and is $\sim 10^{-15}$*

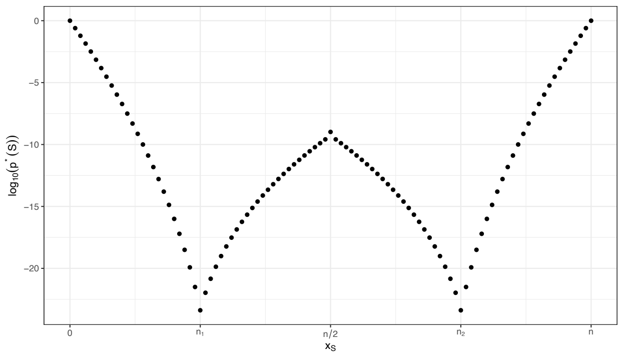

Fig. S2: Inspired from Llinares-López *et al.* (2015) *Minimum p-value as a function of $x_{\mathcal{S}}$ for fixed values of $n_1 = 25$ and $n = 100$. Using the notation from table 1, with $J = 1$, $n_1 = 25$, $n = 100$, the minimal p-value $p^*(\mathcal{S})$ of the χ^2 test is computed for all possible values of $x_{\mathcal{S}}$. For $x_{\mathcal{S}} \geq \max(n_1, n_2)$, the minimal p-value is strictly increasing. If we reach that stage, we can prune the graph and stop the exploration in that direction. Indeed, if $\mathcal{S}' \supseteq \mathcal{S}$ then $x_{\mathcal{S}'} \geq x_{\mathcal{S}}$. So if $p^*(\mathcal{S}) > \frac{\alpha}{k}$, we know that $p^*(\mathcal{S}') > \frac{\alpha}{k}$ without computing it.*

S-5 Benefit of breadth-first search

S-5.1 A simplified scenario

We consider a very simple graph with $p = 3$ nodes, $J = 1$ population and $n = 12$ samples. The graph is displayed in Fig S4a. Using the reduction from `CALDERA`, we generate a tree structure on $\mathcal{C}$, displayed in Fig S4b.

Then we can explore this structure in depth-first or breadth-first, while pruning using $\alpha = 1$. The order resulting from an exploration in depth-first can be found in Table S1 and the order fom the exploration in breadth-first can be found in Table S2. In this simple setting, exploring in breadth only visits 4 subgraphs while exploring in depth visits 7. This is because the BFS enumerates testable subgraphs more quickly, thereby increasing k and lowering the threshold, which means that the branch starting at $\{v_1\}$ is pruned earlier in the exploration.

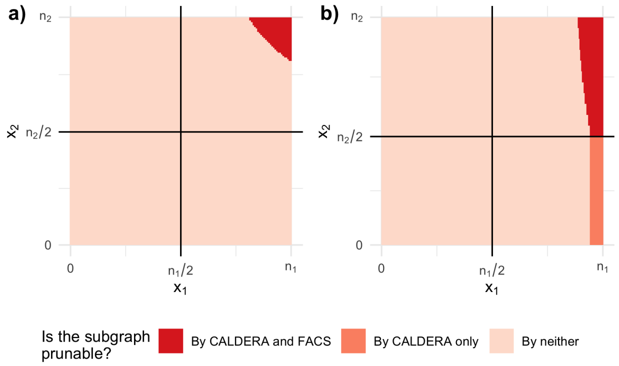

Fig. S3: We consider the space of all possible patterns for $n = 280$, $J = 2$ and two cases: a) $n_1 = n_2 = 140$ and b) $n_1 = 260 = 13 \times n_2$. The phenotypes are well balanced in each population and $alpha = 10^{-8}$. The extended lower bound increases the number of prunable subgraphs when the populations are imbalanced.

Subgraph explored	# of subgraphs explored	Threshold Value	Testable subgraphs
$\{v_1\}$	1	.15	$\{\{v_1\}\}$
$\{v_1, v_2\}$	2	.15/2	$\{\{v_1\}, \{v_1, v_2\}\}$
$\{v_1, v_2, v_3\}$	3	.15/2	$\{\{v_1\}, \{v_1, v_2\}\}$
$\{v_1, v_3\}$	4	.15/3	$\emptyset$
$\{v_2\}$	5	.15/3	$\{\{v_2\}\}$
$\{v_2, v_3\}$	6	.15/3	$\{\{v_2\}, \{v_2, v_3\}\}$
$\{v_3\}$	7	.15/3	$\{\{v_2\}, \{v_2, v_3\}, \{v_3\}\}$

Table S1. Order of exploration of the elements of $\mathcal{C}$ while exploring depth-first

Subgraph explored	# of subgraphs explored	Threshold Value	Testable subgraphs
$\{v_1\}$	1	.15	$\{\{v_1\}\}$
$\{v_2\}$	2	.15/2	$\{\{v_1\}, \{v_2\}\}$
$\{v_3\}$	3	.15/3	$\{\{v_2\}, \{v_3\}\}$
$\{v_2, v_3\}$	4	.15/3	$\{\{v_2\}, \{v_2, v_3\}, \{v_3\}\}$

Table S2. Order of exploration of the elements of $\mathcal{C}$ while exploring breadth-first

S-5.2 A more general setting to understand why BFS is more efficient than DFS

We consider a very simple graph model where, for $v \in \mathcal{V}$ and $i \in \{1, \ldots, n\}$, $i \in \mathcal{I}(v) \sim$ Binom(prop) and the patterns are independent across nodes . We have no population structure, which means that we consider Fisher's exact test. For a given level α, we want to compute $f(\alpha, \text{prop}) = \mathbb{P}(p^\star(\{v\}) > \alpha, \forall v \in \mathcal{V})$, that is the probability that no subgraph is testable at the first stage of our tree on $\mathcal{C}$.

Since we consider Fisher's exact test, there is a bijection between $p^\star(\{v\})$ and $x_{\{v\}}$ so $p^\star(\{v\}) > \alpha \implies x_{\{v\}} \geq \sigma(\alpha)$. Moreover, $x_{\{v\}} \sim \mathcal{B}(\text{prop}, n)$, so $f(\alpha, \text{prop}) = 1 - \left(\mathbf{F}_{\mathcal{B}\rangle\backslash\wr\Updownarrow(\text{prop},n)}(\sigma_\alpha)\right)^p$ with $\mathbf{F}_{\mathcal{B}\rangle\backslash\wr\Updownarrow(\text{prop},n)}$ the cumulative distribution function of the binomial (prop, n). Since the nodes are independent, the distribution of $x_{\mathcal{S}}$ at any stage of the tree can be computed by recursion. We furthermore assume that the graph structure is such that the number of closed subgraphs is $s \times p$ at stage s.

In Fig S4c, we display the probability that any subgraph is 1-testable or prunable at stage s, for $s \in \{1, 2, 3\}$, $p = 100$ and $\alpha = 10^{-4}$.

For most of the range of values, there is at least one testable subgraph in the first stage. So, by exploring in a BFS manner, we start the second stage

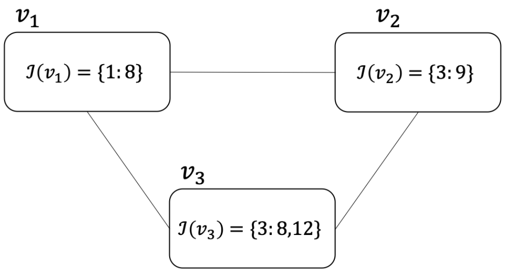

(a) A simple graph example with $p = 3$, $J = 1$ and $N = 12$ which we explore using CALDERA with pruning, and $\alpha = .15$

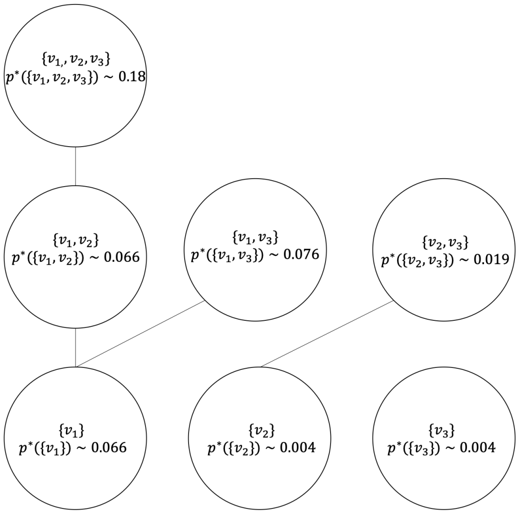

(b) Order on elements of $\mathcal{C}$ from the graph in a), according to the reduction of definition 2

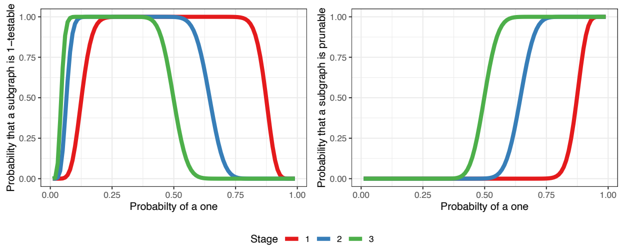

(c) $n = 100, p = 100, \alpha = 10^{-4}$. For a simple model described in S-5.2 where $i \in \mathcal{I}(v) \sim$ Binom(prop), we plot the probability that any subgraph is 1-testable or prunable as a function of prop

Fig. S4: Simple examples where the search in breadth-first is much more efficient that depth-first

with a much lower threshold (i.e., a much higher value of k) which leads to more pruning. For very low values of prop, there might be no testable subgraphs at the first stage but there will be at the second stage, which still justifies an exploration in depth. Note that for large p, we can see that there is no testable subgraph at the stages 2 and 3. That is because all such subgraphs have a pattern that is too large. While there may be not testable subgraphs, there are many prunable ones. In that case, an exploration in breadth-first or depth-first would be identical.

This example simplifies two aspects which have opposite effects. The first is that, in practice, the probability of $i \in \mathcal{I}(v)$ is of course not uniform across the graph. It is a distribution with much heavier tails which means that, even if the average number of 1 might be small, it is still quite likely that at least one subgraph is testable. The second is that the patterns of neighbouring nodes are correlated. As such, the patterns cannot increase by as much between stages, which limits both the increase in testable pattern discovery, and the pruning.

S-6 Speed Simulations

S-6.1 General simulation settings

For given values of n (number of samples) and p (number of nodes), we first generate n samples with phenotype $y_i \in \{0,1\}$ such that $\mathbf{P}(y_i = 0) = prop$ (user defined parameter). Then, we generate p nodes. 10% of the nodes will be associated with the phenotype. For each node in the remaining 90%, we randomly generate 3 edges between this node and another in the 90%. The average degree is therefore 6. For those nodes v_j, the associated pattern $\mathcal{I}(v_j)$ is a random vector such that $\mathbf{P}(i \in \mathcal{I}(v_j)) = 0.5$.

Then, we generate the remaining 10% of the nodes associated with the phenotype. We first generate associated patterns $\mathcal{I}_{sig}$ such that $\mathbf{P}(i \in \mathcal{I}_{sig}|y_i = 1) = 0.95$ and $\mathbf{P}(i \in \mathcal{I}_{sig}|y_i = 0) = 0.05$. Then, those patterns are split into 10 significant nodes sig_j such that $\mathbf{P}(i \in \mathcal{I}(\text{sig}_j)|i \in \mathcal{I}_{sig}) = 0.9$ and $\mathcal{I}(\bigcup_{j\in[1\ldots10]}) = \mathcal{I}_{sig}$.

S-6.2 Parameters for various scenarios

We increase p until COIN+LAMP2 times out (sometimes we went a little further to continue investigation the behaviors).

**Scenario**	1	2	3	4
n	100	50	100	100
$prop$	.5	.5	.2	.2
α	.05	.05	.05	10^{-4}
timeout (days)	2	1	1	1
max value of p	2×10^4	2×10^4	2×10^3	5×10^4

Table S3. Parameter values for the simulations

S-6.3 Results on all scenarios

All computations were run on a r3.4xlarge AWS machine with 16 vCPUs (8 physical ones) and 122GiB(AWS, 2020). We stop every method once it runs for more than timeout. We also stopped running the entire scenario once we have reached timeout for COIN+LAMP2 (expect for scenario 3 where we continued further to study the behavior of the various modes of CALDERA.

S-6.4 Memory requirements

We also launched scenario 2 while monitoring memory usage for COIN+LAMP2, CALDERA BFS and CALDERA DFS. CALDERA DFS uses 1/3 of the peak memory of CALDERA BFS. This is expected since the tree structure that is explored scales in p in breadth but in $n << p$ in depth. Memory-wise, CALDERA BFS is on par with COIN+LAMP2, which relies on a DFS search. This shows that the use of local itemset tables offers memory gains that are enough to offset the exploration in breath, while providing large speed gains. This also suggests that hybrid explorations might be even better at navigating the memory-speed trade-off.

S-6.5 Imbalance

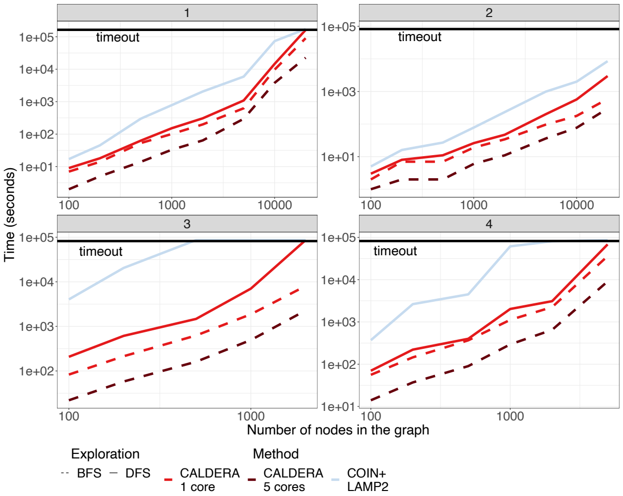

Fig. S5: Runtimes for CALDERA and COIN+LAMP on graphs with various values of covariates p and various values of the simulation parameters.

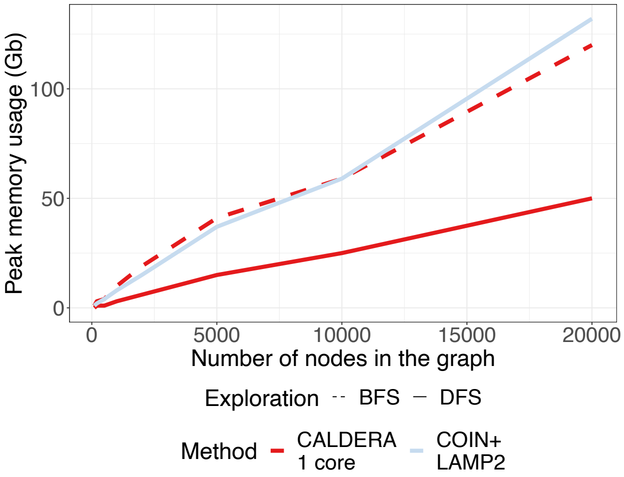

Fig. S6: Peak memory usage for CALDERA and COIN+LAMP on graphs with various values of covariates p.

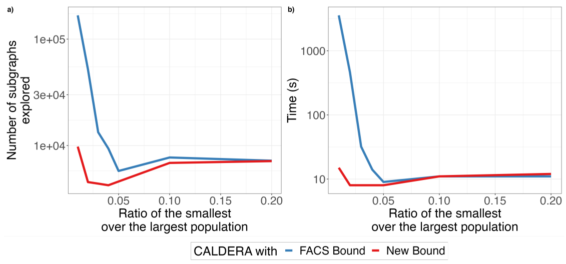

Fig. S7: Impact of the new envelope bound on the number of explored subgraphs **a)** and subsequently on runtime **b)** when there is clear imbalance between the size of the two populations.

S-7 Power Simulations

S-7.1 Data generation

We first generate two sequences of nucleotides of 1000 bps, gene A and gene B. Then, for each gene, we generate 10 versions. For each version, we start from the original copy and introduce mutations in the following manners: at each base, there is a 1% chance of a point mutation (with each mutation equally likely), a 1% chance of a deletion, a 1% chance of a insertion after the base (with each insertion being equally likely) and therefore a 97% chance that nothing happens. We then generate 50 sensitive samples by randomly selecting with replacement a version of gene B: this is their entire genome. We also generate 50 resistant samples by randomly selecting with replacement a version of gene A and a version of gene B and collating them together. We therefore obtain 100 genetic sequences, 50 of each phenotype.

S-7.2 Testing all methods

We build a De Bruijn Graph of 704 unitigs using the first step of `DBGWAS`. Then, we run `DBGWAS` with the parameter *-SFF q1.0* to avoid filtering any unitig and then keep those whose p-values are smaller than $\alpha/704$. We also tests the pattern of each unitig for association with the vector of phenotypes. We use the `LAMP2` procedure to remove non-testable hypotheses, at levels of α. Finally, we run `CALDERA` with options *–Lmax=2000 -C 1*, and we either specify the maximum number of stages (among $[1, 2, 3, 5, 10, 15, 20]$) or we run all stages. We look at the list of significant CCSs and keep all their unitigs as significant.

S-7.3 Results

We compute two metrics: the proportion of unitigs called significant divided by all the unitigs that originated from gene A, named **coverage**; and the proportion of unitigs called significant divided by all the unitigs that originated from gene B, named **False Positive Rate**. We can plot those values for all three methods, and over all ranges of the parameter *–stage* for `CALDERA`

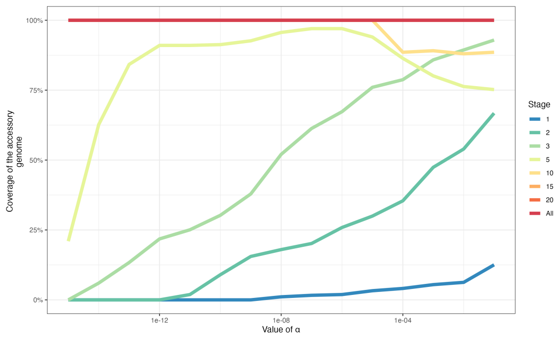

Fig. S8: Proportion of all unitigs associated with the resistant phenotype that are found to be significant by `CALDERA` when varying the maximum number of stages explored.

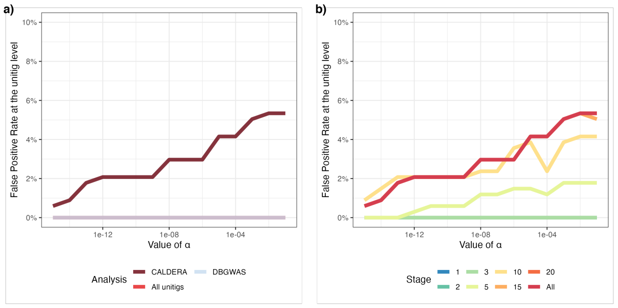

Fig. S9: Proportion of all unitigs not associated with the resistant phenotype that are found to be significant as the value of α changes **a)** by `CALDERA`, the `LAMP2` procedure on all unitigs and `DBGWAS`, and **b)** by `CALDERA` when varying the maximum number of stages explored.

Figure S11 shows the computational ressources used by `CALDERA` against the BFS stage. Memory usage is exponential in the BFS stage, which is consistent with the number k_0 of explored CCS (not shown), but as seen above, this does not reflect the final coverage of the compacted DBG by significant CCS. The elapsed time increases linearly, with some variation that can be explained by external factors impacting the computation nodes. We do not report the results for stage 8 on the same plot as they were done with a smaller batch size of $100,000$ and are therefore not comparable: the corresponding run used 947Gb of RAM and took 44h30.

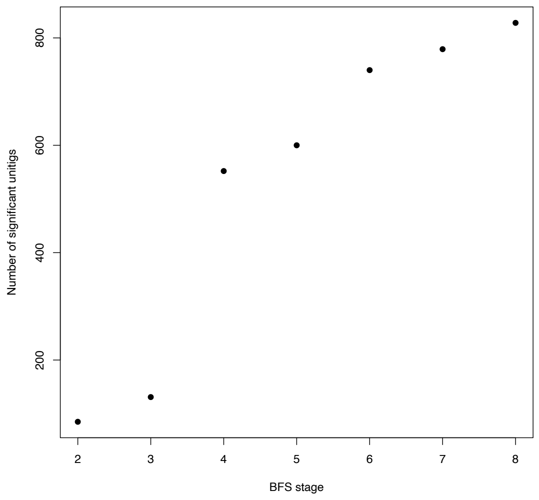

Fig. S10: Number of unitigs belonging to at least one significant CCS for increasing BFS stages on the **Pseudomonas** dataset with $\alpha = 10^{-6}$.

S-8 Behavior of CALDERA against BFS stages

Figure S10 shows the number of unitigs belonging to at least one significant CCS for increasing BFS stages on the **Pseudomonas** dataset with $\alpha = 10^{-6}$. This number increases sharply at stage 4, where the large CCS containing the plasmid are found, then increases more slowly and reaches a plateau after stage 6.

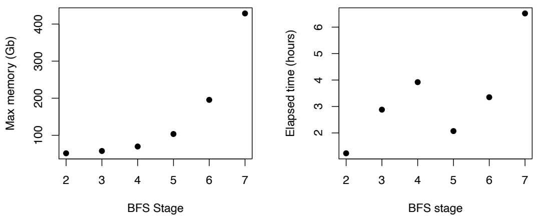

Fig. S11: Computational ressources used for increasing BFS stages on the **Pseudomonas** dataset with $\alpha = 10^{-6}$, using a batch size of $200,000$ and four cores. Left panel: peak memory used during computation in Gb. Right panel: elapsed time in hours.

S-9 The plasmid, returned as the first component by CALDERA when run on the Pseudomonas dataset.

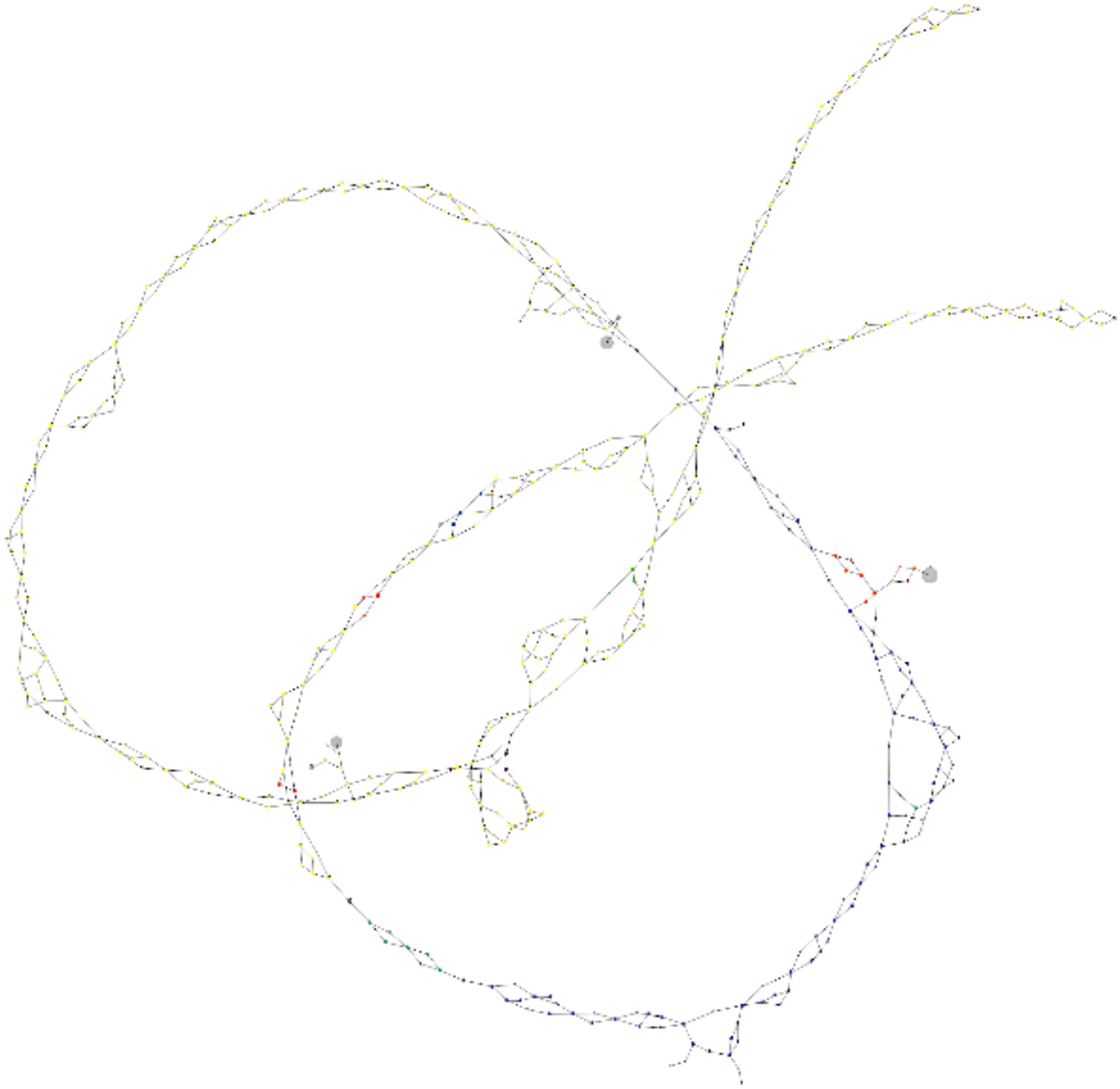

Fig. S12: **Pseudomonas** dataset: plasmid

Fig. S13: Screenshot from the output of `CALDERA`. We select the first component, that is the one which contains the most significant CCS. Unitigs belonging to the same CCS are colored in the same way. If two significant CCSs are less than two unitigs apart, they are represented in the same component.

S-10 Network-guided GWAS on *A. thaliana* genomes

We obtained over 6 millions SNPs and a "date to flowering" phenotype for $n = 936$ *A. thaliana* genomes from easyGWAS (Grimm *et al.*, 2017). We also obtained 137 *A. thaliana* metabolic pathways from KEGG (Kanehisa and Goto, 2000) using the `KEGGrest` (Tenenbaum, 2020) and `DEGraph` (Jacob *et al.*, 2012) R packages. The union of the pathways involved $p = 3150$ genes and the average degree of the resulting graph is ~ 21.2. We mapped each SNP to the closest gene using `snpEff` (Cingolani *et al.*, 2012) and defined each gene to be mutated in a sample if it contained at least one mutation mapping to the gene. Runtime was under a minute using 8 cores. $k_0 = 70$ and 10 subgraphs are found to be significant. In particular, the first and second most significant subgraphs involve pathways `ATH00260`:*Glycine, serine and threonine metabolism* and `03013`: *RNA transport*, which were previously linked to flower development (Hesse and Hoefgen, 2003; Pfaff *et al.*, 2018).
